# CHAC2-mediated glutathione metabolic reprogramming drives N1 polarization of bone marrow neutrophils and exacerbates inflammatory comorbidities

**DOI:** 10.1038/s41368-026-00451-6

**Published:** 2026-07-09

**Authors:** Yuting Niu, Yuman Li, Shiyu Sun, Chenyu Deng, Fan He, Yiming Chen, Tiansong Xu, Zhewen Hu, Gai Yang, Leran Li, Boon Chin Heng, Yan Lv, Ying Huang, Xuliang Deng

**Affiliations:** 1 Department of Geriatric Dentistry, Peking University School and Hospital of Stomatology & National Center for Stomatology & National Clinical Research Center for Oral Diseases & National Engineering Research Center of Oral Biomaterials and Digital Medical Devices, Beijing, China; 2 Department of Orthodontics, Peking University School and Hospital of Stomatology & National Center for Stomatology & National Clinical Research Center for Oral Diseases & National Engineering Research Center of Oral Biomaterials and Digital Medical Devices, Beijing, China; 3 Fifth Clinical Division, Peking University School and Hospital of Stomatology & National Center for Stomatology & National Clinical Research Center for Oral Diseases & National Engineering Research Center of Oral Biomaterials and Digital Medical Devices, Beijing, China; 4 Central Laboratory, Peking University School and Hospital of Stomatology, Beijing, China; 5 Beijing Institute of Dental Research, Beijing Stomatological Hospital, Capital Medical University, Beijing, China

**Keywords:** Periodontitis, Diabetes

## Abstract

The proinflammatory (N1) polarization of bone marrow (BM) neutrophils, driven by central immune remodeling in response to peripheral inflammation, plays a critical role in propagating localized inflammatory conditions, such as periodontitis, to systemic levels. Although this process involves metabolic reprogramming, the specific underlying metabolic mechanisms of neutrophil N1 polarization within the periodontitis-modified BM niche remain poorly defined. Integrated transcriptomic and metabolomic analyses in this study revealed that periodontitis reprograms intracellular glutathione (GSH) metabolism in BM neutrophils, facilitating their N1 polarization. Central to this mechanism is the upregulation of Chac2, an enzyme that promotes GSH accumulation. This enhancement is accompanied by elevated GSH redox cycling, which supports sustained ROS production and NET formation, thereby amplifying inflammatory responses. We further identified type I interferon (IFN-I) signaling as a key upstream regulator that induces Chac2 expression and drives metabolic reprogramming. Importantly, the intraosseous delivery of AAV-delivered Chac2 shRNA in *db/db* mice with periodontitis markedly reduced neutrophil-aggravated systemic inflammatory comorbidity symptoms and improved glycemic control, underscoring the functional relevance of this pathway in diabetic comorbidity. Together, these findings thus delineate the IFN-I–Chac2–GSH axis as a core signaling mechanism regulating neutrophil N1 polarization in the BM niche, providing new insights into how periodontal inflammation reprograms immune functions at the systemic level. This study thus broadens the conceptual framework of neutrophil immunometabolism and proposes targeting the Chac2–GSH axis as a potential therapeutic strategy for systemic comorbidities associated with periodontitis.

## Introduction

Local chronic inflammation not only extends beyond a pathological state localized within specific tissues/organs, but also profoundly reshape systemic immunity through peripheral inflammatory signaling.^1–4^ In this process, the bone marrow (BM), as the central tissue/organ of hematopoiesis, undergoes marked alterations in hematopoietic dynamics, manifested by skewed myeloid differentiation and increased neutrophil production.^5^ Our previous study had revealed that this quantitative expansion is accompanied by the functional activation of neutrophils, characterized by enhanced release of reactive oxygen species (ROS), increased formation of neutrophil extracellular traps (NETs), and elevated secretion of pro-inflammatory cytokines such as tumor necrosis factor-α (TNF-α).^6^ Collectively, these functional changes are consistent with neutrophil polarization toward the N1 proinflammatory phenotype,^7^ underscoring the propensity of neutrophils to undergo such polarization within the BM microenvironment remodeled by chronic inflammation. Immune cell polarization is intricately linked to underlying metabolic reprogramming,^8–11^ which serves as a critical determinant of cellular phenotype and effector activity.^12–14^

The intrinsic connection between immune cell polarization and metabolic reprogramming has emerged as a central theme in immunometabolism research, offering critical insights into the regulatory mechanisms of immune responses. Indeed, substantial evidence has demonstrated that specific metabolic reprogramming directly regulates immune cell function, providing new perspectives and potential targets for the metabolic modulation of immune-mediated diseases. This principle has been validated across multiple immune cell types. For example, glycolytic metabolism supports macrophage M1 polarization,^9,10,15,16^ lipid metabolism governs T cell differentiation,^17–20^ and mitochondrial dynamics influence dendritic cell activation.^21–24^ Given the robust evidence for these conserved mechanisms, growing research attention is now directed towards the metabolic adaptations of neutrophils,^11,25,26^ particularly how these immune cells remodel their metabolism in response to specific pathological microenvironments.

In the tumor microenvironment, neutrophils have been shown to undergo glycolytic reprogramming in response to inflammatory cytokines, driving their polarization toward an N1 phenotype that exerts anti-tumor functions.^27,28^ However, it is still unclear whether neutrophils experience comparable metabolic adaptations within the BM microenvironment remodeled by chronic inflammation (such as periodontitis). More importantly, how such metabolic remodeling directly governs the process of N1 polarization in this context is still unresolved, representing a critical knowledge gap on how metabolic regulation is linked to neutrophil inflammatory phenotypes.

Among the various diverse pathways of immunometabolic regulation, the metabolism of glutathione (GSH), a key endogenous antioxidant, plays key roles in maintaining the redox homeostasis, survival, and effector functions of immune cells.^29,30^ Its intracellular levels are precisely regulated by a metabolic network that includes the synthetic enzyme Gss,^31^ the degradation enzyme Chac1, and the degradation regulator Chac2, which work together to sustain GSH metabolic balance.^30,32^ The immunoregulatory role of GSH biosynthesis mediated by key enzymes such as Gclc and Gss has been well established in the regulation of macrophage inflammatory activation, monocyte redox-dependent cytokine production, and T cell differentiation.^33–37^ However, the contribution of GSH degradation pathway—particularly the regulatory functions of Chac2 under pathological conditions—remains poorly understood. It is necessary to investigate whether neutrophils can undergo Chac2-mediated GSH metabolic reprogramming within the BM niche remodeled by chronic inflammation, which could drive their polarization toward the N1 state. In this study, a murine periodontitis model was used to investigate how local chronic inflammation triggers neutrophil proinflammatory N1 polarization within the BM niche. By integrating transcriptomic and untargeted metabolomic analyses of BM neutrophils, we revealed a pivotal role for Chac2-driven GSH metabolic reprogramming in skewing neutrophils toward the N1 phenotype, which is orchestrated by upstream IFN-I signaling. By further investigating the systemic impact of this polarization in diabetic mice and evaluating the effects of Chac2 inhibition, we provide mechanistic insights into the underlying metabolic mechanisms of neutrophil N1 polarization, which opens up a new therapeutic avenue for targeting BM neutrophil-driven comorbidities.

## Results

### Periodontitis promotes N1-type polarization of BM neutrophils through enhanced GSH metabolism

**Fig. 1:**
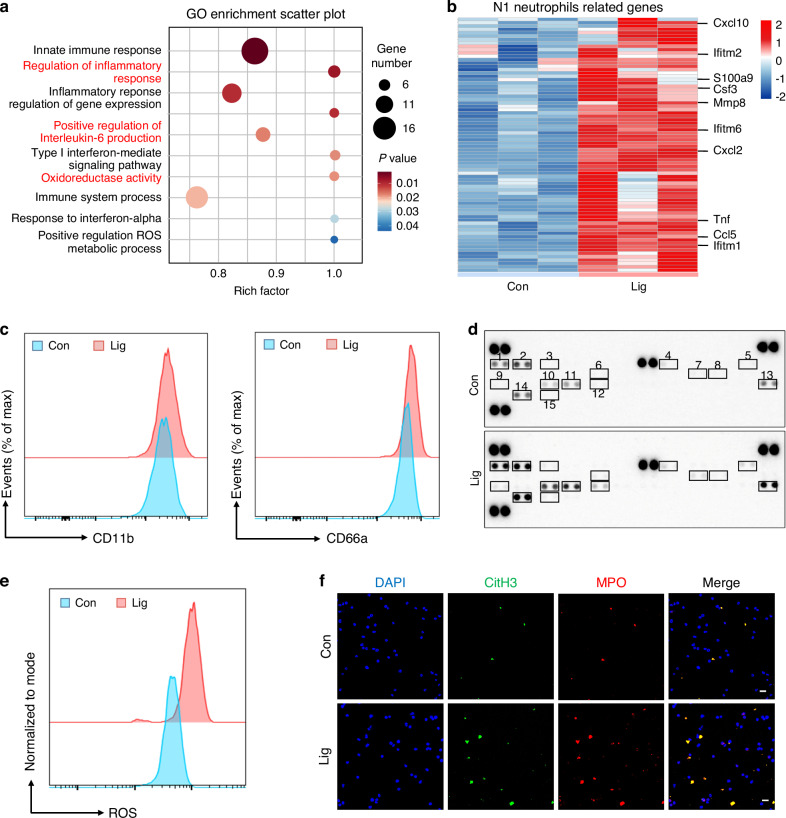
Periodontitis induces N1-type polarization of BM neutrophils. **a** GO enrichment analysis for genes differentially upregulated in the Lig group compared to the Con group. **b** Heat map of the expression of N1 neutrophil-related genes. **c** Flow cytometric analysis of the expression of CD11b and CD66a in neutrophils obtained from the BM. **d** Cytokine antibody array dot plot of differentially-expressed cytokines produced by neutrophils. **e** Overlay histogram of ROS production in neutrophils. **f** Representative images of neutrophils stained with citH3 (green), MPO (red), and DAPI (blue). NETs are visualized by co-localization of citH3 and DAPI staining (merged images), scale bar = 10 µm

To investigate the impact of periodontitis on the polarization state of BM neutrophils, we analyzed RNA-seq data from BM neutrophils of the control (Con) and ligature-induced periodontitis (Lig) mice obtained in our previous study.^6^ Gene Ontology (GO) analysis of differentially expressed genes revealed enrichment of pathways related to neutrophil activation and inflammation, including “innate immune response”, “regulation of inflammtory response” and “positive regulation of chemokine production” (Fig. 1a). Notably, BM neutrophils of Lig group exhibited upregulated expression of representative N1-type markers^7^ such as S100a9, Ifitm1, Ifitm2, and Cxcr10 (Fig. 1b), and flow cytometry (FCM) confirmed elevated surface levels of CD11b and CD66a (Fig. 1c and Fig S1a). To mimic the inflammatory environment that pre-activated BM neutrophils encounter in peripheral tissues, Con and Lig BM neutrophils were stimulated with LPS ex vivo. Consequently, these cells displayed heightened inflammatory activities, including increased cytokine release (Fig. 1d and Fig S1b), superior ROS generation (Fig. 1e and Fig S1c), and more robust NETs (Fig. 1f and Fig S1d). These results thus indicate that periodontitis drives BM neutrophil polarization towards a proinflammatory N1 phenotype at the transcriptional, phenotypic, and functional levels.

**Fig. 2:**
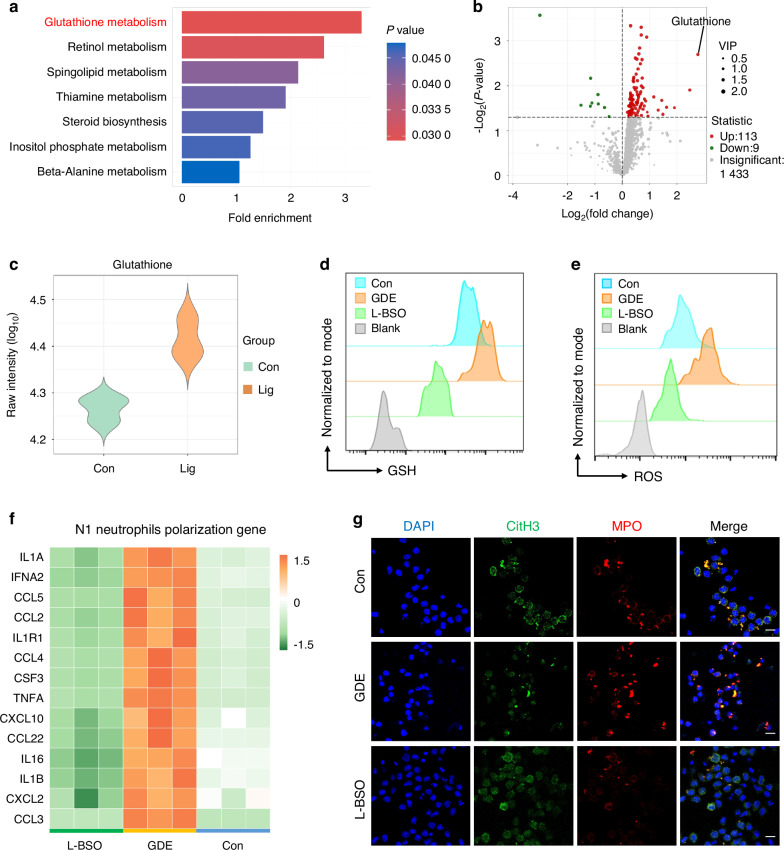
GSH accumulation is associated with N1-type neutrophil activation. **a** KEGG pathway enrichment analysis of metabolites differentially upregulated in the Lig group versus the Con group. **b** Volcano plot analysis identifying the most differentially expressed metabolite between the Lig and Con groups. **c** Violin plot comparing the GSH quantities of the Con group versus the Lig group in the raw intensity of metabolomic data. **d** Overlay histogram of GSH concentration in Con, GDE and L-BSO neutrophils. **e** Overlay flow cytometry histograms of intracellular ROS levels in Con, GDE and L-BSO neutrophils. **f** Heat map of gene expression related to N1-type neutrophil polarization. **g** Representative immunofluorescent staining of CitH3 (green), MPO (red), and cell nuclei (DAPI, blue) of neutrophils. Scale bar = 10 μm

Given the critical role of metabolism in shaping immune cell function, we next examined whether N1 polarization was accompanied by alterations in metabolic pathways. Untargeted metabolomic profiling of sorted BM neutrophils revealed a distinct separation between the Con and Lig groups by PLS-DA with 122 metabolites significantly altered, among which amino acid derivatives were most enriched (Fig S2a–c). Both KEGG and MSEA pathway analyses identified glutathione metabolism as the top-enriched pathway (Fig. 2a and Fig S2d). Notably, intracellular GSH levels were elevated nearly fourfold in Lig-derived neutrophils (Fig. 2b, c), which was further validated by ELISA quantification (Fig S2e).

To functionally assess the role of GSH in driving neutrophil polarization, we utilized the HL60 cells differentiated into neutrophil-like cells. Cells were treated with either glutathione diethyl ester (GDE) to enhance intracellular GSH or L-buthionine sulfoximine (L-BSO) to inhibit GSH synthesis. GSH levels were confirmed by FCM using a GSH-sensitive probe (Fig. 2d and Fig S2f). GDE-treated cells exhibited upregulation of N1 markers, along with increased NETs formation, ROS production, and proinflammatory cytokine secretion, all of which were significantly suppressed by L-BSO treatment (Fig. 2e–g and Fig S2g–j). Consistently, primary mouse BM neutrophils treated by GDE or L-BSO treatment showed parallel changes in intracellular GSH and ROS levels, as well as in the production of inflammatory cytokines (Fig S2k–m).

Together, these results thus demonstrate that periodontitis promotes N1-type polarization of BM neutrophils and is closely associated with enhanced GSH metabolic activity, implicating GSH metabolism as a key regulatory axis in inflammation-driven neutrophil reprogramming.

### Periodontitis induces glutathione metabolic reprogramming in BM neutrophils

**Fig. 3:**
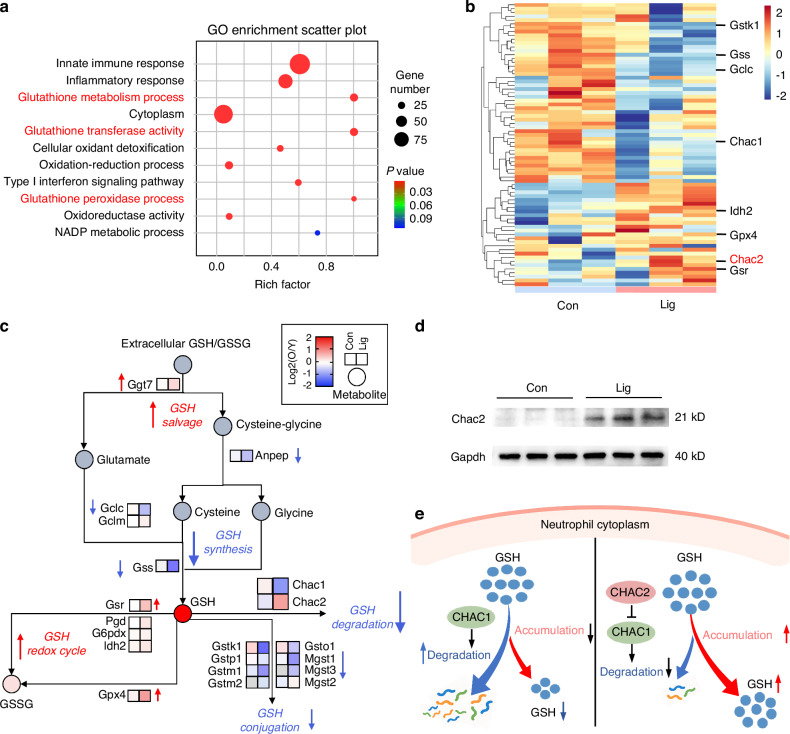
GSH metabolic reprogramming in BM neutrophils is driven by CHAC2-mediated inhibition of its degradation. **a** GO enrichment analysis of genes differentially upregulated in the Lig group versus the Con group. **b** Heat map of gene expression related to the GSH metabolic pathway. **c** Diagram showing the up-regulation (red) and down-regulation (blue) of key metabolites and key genes in the GSH metabolic pathway. **d** Western blotting and quantitative analyses of Chac2 protein expression levels in BM neutrophils from Con versus Lig mice. **e** Schematic diagram illustrating the regulatory role of CHAC2 in GSH degradation

To investigate how periodontitis alters GSH metabolism in BM neutrophils, we conducted an integrated analysis of transcriptomic and metabolomic datasets from Con and Lig mice. GO enrichment analysis of genes upregulated in Lig mice identified significant activation of the “glutathione metabolic process” (Fig. 3a). Differential gene expression profiling revealed notable changes in key glutathione-related genes (Fig. 3b).

Subsequently, an integrated GSH metabolic network map based on both datasets was constructed, which revealed a distinct reprogramming pattern in the Lig group. Despite elevated intracellular GSH levels and increased redox cycling activity, the transcriptomic data indicated downregulation of Gss (glutathione synthetase), Gclc (glutamate cysteine ligase catalytic), and Chac1 (a canonical GSH-degrading enzyme), alongside significant upregulation of Chac2, a less characterized homolog involved in glutathione regulation (Fig. 3c).

Given that previous studies have shown that Chac2 can functionally antagonize Chac1-mediated GSH degradation, likely due to its slower catalytic turnover and distinct substrate-binding properties, and supports a model in which CHAC2 competitively binds reduced GSH but degrades it inefficiently, thereby favoring net GSH retention.^38–40^ This hypothesis was supported by altered total GSH concentration, GSH/GSSG ratio, and RT-qPCR validation of gene expression patterns in Con and Lig neutrophils (Fig S3a–f). Western blot analysis further confirmed a ~ 3.5-fold increase in Chac2 protein expression in Lig samples compared to controls (Fig. 3d and Fig S3g). The expression of Gsr and Gpx4 increased at transcriptional level, and the enzymatic activity of neutrophils in the Lig group was enhanced (Fig S3h), suggesting that the accumulation of GSH within cells was accompanied by an increase in the redox cycle.

Collectively, these findings suggest that periodontitis induces a distinct reprogramming of GSH metabolism in BM neutrophils, characterized by CHAC2-mediated suppression of GSH degradation (Fig. 3e), which may play a pivotal role in modulating neutrophil polarization.

### Chac2-mediated GSH metabolic reprogramming drives N1-type polarization of neutrophils

**Fig. 4:**
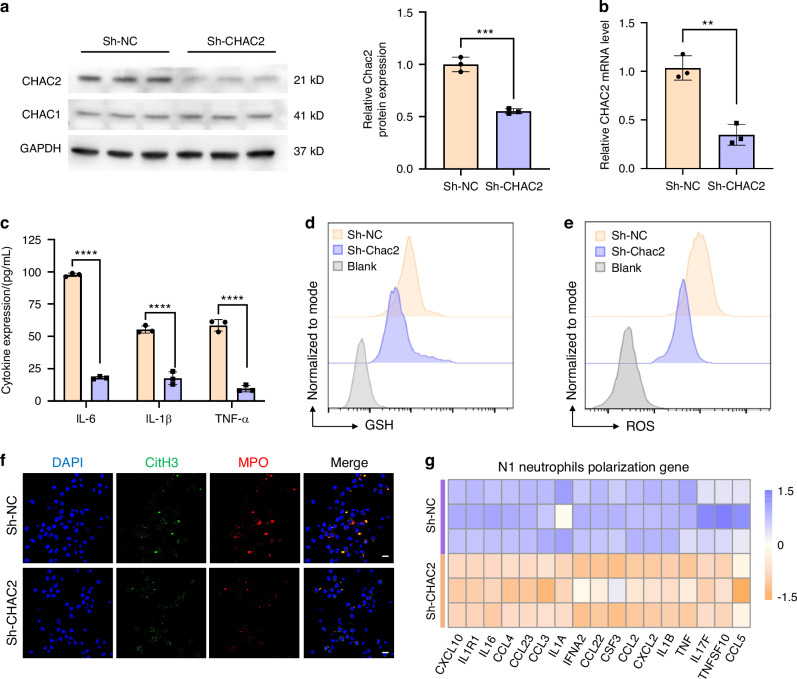
Knockdown of CHAC2 attenuates neutrophil N1 polarization by reducing GSH accumulation. **a** Western blotting and quantitative analyses of CHAC2 and CHAC1 protein expression levels in neutrophils of Sh-NC and Sh-CHAC2 groups. **b** Statistical chart of relative changes in *CHAC2* mRNA expression levels. **c** Quantitative statistical analyses of the expression levels of cytokines IL-6, IL-1β and TNF-α produced by neutrophils. **d** Overlay histogram of GSH concentration in neutrophils. **e** Overlay histogram of ROS production. **f** Representative immunofluorescent staining of CitH3 (green), MPO (red), and cell nuclei (DAPI, blue) of HL60 neutrophils. Scale bar = 10 μm. **g** Heat map of PCR array-based gene expression analysis related to N1-type polarization of Sh-NC and Sh-Chac2 neutrophils. Data are presented as the mean ± SD from at least three independent experiments. The *P* values were calculated using two-tailed Student’s *t*-test (**a**, **b**) and two-way ANOVA followed by Bonferroni’s multiple comparisons test (**c**); *P < 0.05, **P < 0.01, ***P < 0.001, ****P < 0.000 1, ns indicates no significant difference

To determine whether Chac2 is functionally involved in GSH-driven N1-type polarization, we performed both loss- and gain-of-function experiments using HL60-derived neutrophils. Knockdown of Chac2 (Sh-Chac2) via lentiviral transduction significantly reduced its expression at both the mRNA and protein levels, as confirmed by RT-qPCR and Western blot (Fig. 4a, b). Chac2 silencing led to a marked decrease in intracellular GSH content, accompanied by significantly reduced levels of proinflammatory cytokines, ROS production, NETs formation, and suppressed expression of N1 signature genes (Fig. 4c–g and Fig S4a–c). These results thus indicate that Chac2 knockdown impairs GSH accumulation and attenuates N1-type inflammatory polarization.

**Fig. 5:**
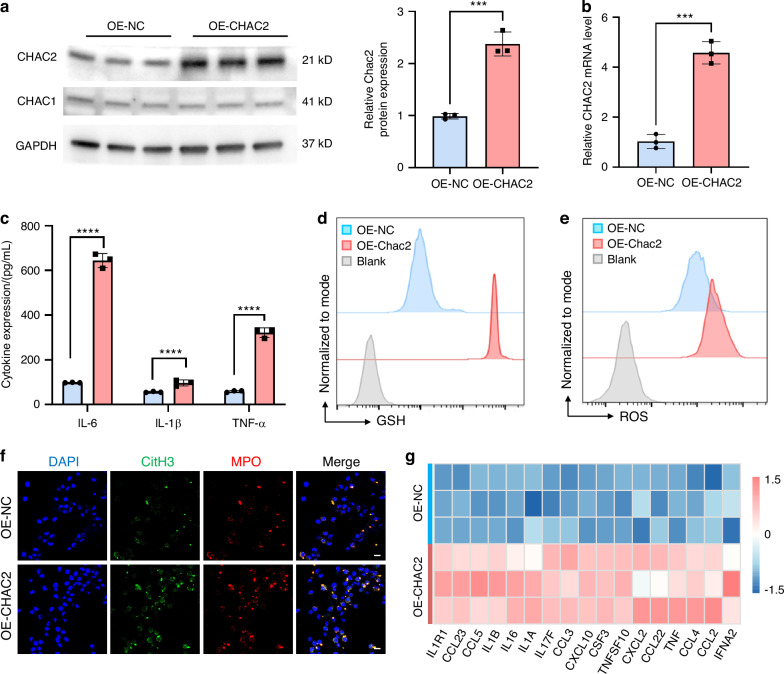
CHAC2 serves as a key metabolic regulator linking GSH homeostasis to neutrophil polarization. **a** Western blotting and quantitative analyses of CHAC2 and CHAC1 protein expression levels in neutrophils of OE-NC and OE-CHAC2 groups. **b** Statistical chart of relative changes in *CHAC2* mRNA expression levels. **c** Quantitative statistical analyses of the expression levels of cytokines IL-6, IL-1β, and TNF-α produced by neutrophils. **d** Overlay histogram of GSH concentration in neutrophils. **e** Overlay histogram of ROS production. **f** Representative immunofluorescent staining of CitH3 (green), MPO (red), and cell nuclei (DAPI, blue) of neutrophils. Scale bar = 10 μm. **g** Heat map of PCR array-based gene expression analysis related to N1-type polarization of OE-NC and OE-Chac2 neutrophils. Data are presented as the mean ± SD from at least three independent experiments. The *P* values were calculated using two-tailed Student’s *t*-test (**a**, **b**) and two-way ANOVA followed by Bonferroni’s multiple comparisons test (**c**); *P < 0.05, **P < 0.01, ***P < 0.001, ****P < 0.000 1, ns indicates no significant difference

Conversely, the overexpression of Chac2 (OE-Chac2) in HL60 cells (Fig. 5a, b) resulted in a robust increase in GSH levels, validating its role in maintaining GSH homeostasis. Functionally, Chac2 overexpression led to enhanced secretion of IL-6, TNF-α, and IL-1β, elevated ROS production, increased NET formation, and significant upregulation of N1-associated genes (Fig. 5c–g and Fig S5a–c), collectively indicating a shift towards a pro-inflammatory N1 phenotype.

Together, these results thus demonstrate that Chac2 serves as a key metabolic regulator linking homeostasis to neutrophil functional polarization. Specifically, Chac2 promotes GSH accumulation and drives N1-type polarization, thus contributing to the inflammatory potential of neutrophils in the context of periodontitis.

### IFN-I signaling promotes Chac2-mediated GSH reprogramming and N1-type polarization of neutrophils

To identify upstream regulatory pathways responsible for the observed GSH metabolic reprogramming in BM neutrophils during periodontitis, we performed gene set enrichment analysis (GSEA) of neutrophil transcriptomes from Lig and Con mice. GSEA analysis revealed significant enrichment of IFN-I signaling pathways in the Lig group (Fig S6a), while the corresponding heatmap analysis confirmed upregulation of IFN-I–responsive genes in Lig-derived neutrophils (Fig S6b). Consistently, the IFN-I levels in BM supernatants were markedly elevated in periodontitis mice (Fig S6c–d), indicating robust activation of IFN-I signaling during periodontitis.

To functionally evaluate the role of IFN-I signaling, we subjected both *WT* and *Ifnar1*^*−/−*^ mice (lacking the type I interferon receptor) to ligature-induced periodontitis. In *Ifnar1*^*−/−*^ mice, periodontitis failed to induce the metabolic and phenotypic changes observed in *WT* animals. Specifically, intracellular GSH levels (Fig S6e) and Chac2 expression (Fig S6f) remained unchanged in Lig-Ifnar1^−/−^ neutrophils compared to controls. Additionally, the key pro-inflammatory features of N1 polarization—including ROS generation (Fig S6g) and proinflammatory cytokine secretion (Fig S6h)—were significantly attenuated. These findings thus suggest that IFN-I signaling is required for Chac2 induction and GSH accumulation, as well as for driving the N1-type pro-inflammatory programming of neutrophils in periodontitis.

**Fig. 6:**
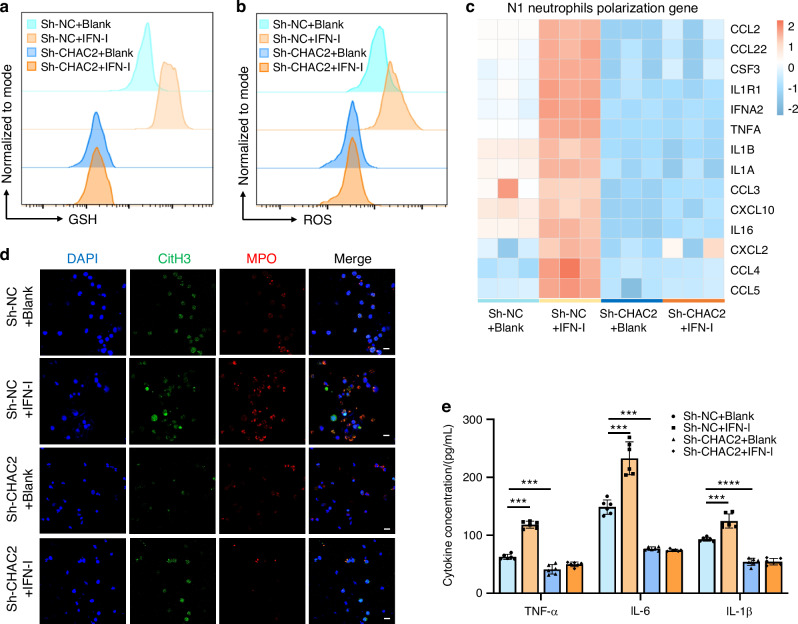
IFN-I signaling promotes proinflammatory N1-type neutrophil polarization by inducing Chac2 expression and modulating GSH metabolism. **a** Overlay histogram of GSH concentrations in Sh-NC and Sh-CHAC2 neutrophils of the blank or IFN-I treatment groups. **b** Overlay histogram of ROS production in Sh-NC and Sh-CHAC2 neutrophils of the blank or IFN-I treatment groups. **c** Heat map of gene expression related to N1 neutrophil polarization. **d** Representative immunofluorescent staining of CitH3 (green), MPO (red), and cell nuclei (DAPI, blue) of neutrophils. Scale bar = 10 μm. **e** Quantitative analysis of the expression levels of the reactive cytokines IL-6, IL-1β, and TNF-α produced by neutrophils. Data are presented as the mean ± SD from at least three independent experiments. *P* values were calculated using one-way ANOVA followed by Tukey’s multiple comparisons test; *P < 0.05, **P < 0.01, ***P < 0.001, ****P < 0.000 1, ns indicates no significant difference

To further delineate the IFN-I–CHAC2 signaling axis in neutrophil polarization, we treated HL60 neutrophils with recombinant IFN-I in the presence or absence of Chac2 knockdown. In Sh-NC cells, IFN-I stimulation induced a robust increase in intracellular GSH levels, upregulated Chac2 expression, and enhanced transcription of N1-type signature genes (Fig. 6a, b and Fig S6i, j). Functionally, IFN-I also promoted NETs formation, ROS production, and pro-inflammatory cytokine release (Fig. 6c–e and Fig S6k–l). However, in Sh-Chac2 cells, these IFN-I–induced effects were markedly ablated—demonstrating that Chac2 is a key mediator linking IFN-I signaling to GSH metabolic reprogramming and N1-type polarization. Hence, these results validated that IFN-I signaling promotes proinflammatory N1-type polarization of neutrophils by inducing Chac2 expression and modulating GSH metabolism.

### AAV-shChac2 delivery alleviated systemic inflammation and improved metabolic outcomes in diabetic mice

Based on the well-established clinical and experimental association between periodontitis and diabetes,^41–47^ diabetes was used as a representative comorbidity model to assess the systemic impact of periodontitis-induced neutrophil reprogramming. To determine whether BM neutrophils activated by periodontal inflammation exacerbate metabolic dysfunction and distal organ injury, we used *db/db* mice as a type 2 diabetes model and wild-type (WT) mice as non-diabetic controls. Both strains underwent maxillary molar ligation to establish a periodontitis–diabetes comorbidity model (DB-Lig) (Fig S7a). Compared with diabetic controls without ligation (DB-Con), DB-Lig mice showed worse glycemic control and more severe pancreatic dysfunction, as demonstrated by random blood glucose, glycated serum protein, intraperitoneal glucose tolerance test (IPGTT), insulin tolerance test (ITT), and pancreatic insulin/glucagon immunofluorescence staining (Fig S7b–e). In addition, MPO immunohistochemistry and FCM revealed increased pancreatic neutrophil (CD11b⁺Ly6G⁺) infiltration in DB-Lig mice (Fig S7f, g). These findings suggest that periodontitis aggravates diabetic pancreatic injury, at least partly through neutrophil activation.

To further test whether this effect is mediated by periodontitis-induced N1-polarized BM neutrophils, BM neutrophils from control and ligatured WT donor mice were adoptively transferred into *db/db* recipient mice (Con-Neus and Lig-Neus) (Fig S8a). CFSE labeling confirmed that transferred neutrophils homed to and were retained in the pancreas (Fig S8b). Compared with Con-Neus mice, Lig-Neus recipients exhibited higher random blood glucose and glycated serum protein levels, impaired glucose tolerance, reduced insulin sensitivity, disrupted islet function, and increased pancreatic neutrophil infiltration (Fig S8c–g). Together, these results indicate that periodontitis-induced BM N1-type neutrophils exacerbate pancreatic dysfunction in diabetes.

**Fig. 7:**
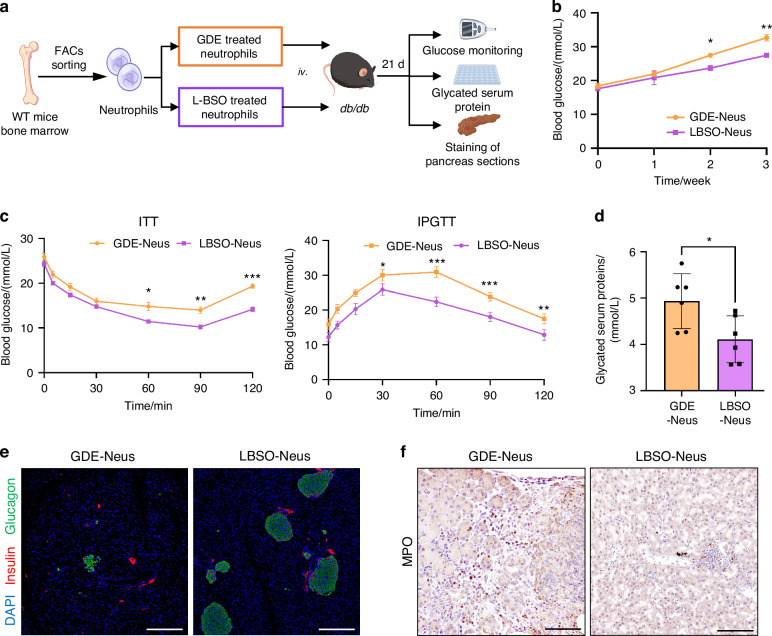
GSH accumulation in neutrophils exacerbates systemic inflammatory injury. **a** Schematic illustration of the experimental design for the transfer of GDE or L-BSO treated BM neutrophils to *db/db* mice (*n* = 6 per group). **b** Statistical curves of random blood glucose measurements in the GDE-Neus and LBSO-Neus groups. **c** Statistical curves of blood glucose measurements during ITT and IPGTT. **d** Quantitative analysis of serum glycated protein content. **e** Representative immunofluorescent staining of glucagon (green), insulin (red), and cell nuclei (DAPI, blue). Scale bar = 200 μm. **f** Immunohistochemical staining of MPO in pancreas tissue sections. Scale bar = 100 μm. All data are presented as the mean ± SD. The *P* values were calculated using two-tailed Student’s t test; *P < 0.05, **P < 0.01, ***P < 0.001, ****P < 0.000 1, ns indicates no significant difference

To directly assess the role of GSH-driven neutrophil N1 polarization in systemic metabolic dysfunction, we adoptively transferred BM neutrophils pre-treated with either GDE (GDE-Neus) or L-BSO (LBSO-Neus) into *db/db* diabetic mice (Fig. 7a). Compared with LBSO-Neus recipients, mice receiving GDE-treated neutrophils, characterized by elevated intracellular GSH and N1-type polarization, exhibited significantly increased random blood glucose and glycated serum protein levels, together with more severe impairment in glucose tolerance and insulin sensitivity, as demonstrated by IPGTT and ITT (Fig. 7b–d). Immunofluorescence staining of pancreatic islets revealed marked insulin depletion and enhanced glucagon expression, indicating disrupted endocrine function (Fig. 7e). MPO immunohistochemistry and FCM analysis showed that neutrophil infiltration in pancreatic tissue was markedly increased in the GDE-Neus group compared with the LBSO-Neus group (Fig. 7f and Fig S9a). Conversely, LBSO-Neus cells with suppressed GSH synthesis and attenuated inflammatory polarization failed to induce these metabolic abnormalities. These results thus validated that the core mechanism by which pro-inflammatory neutrophils exacerbate pancreatic dysfunction in diabetes is intracellular GSH accumulation within the neutrophils.

**Fig. 8:**
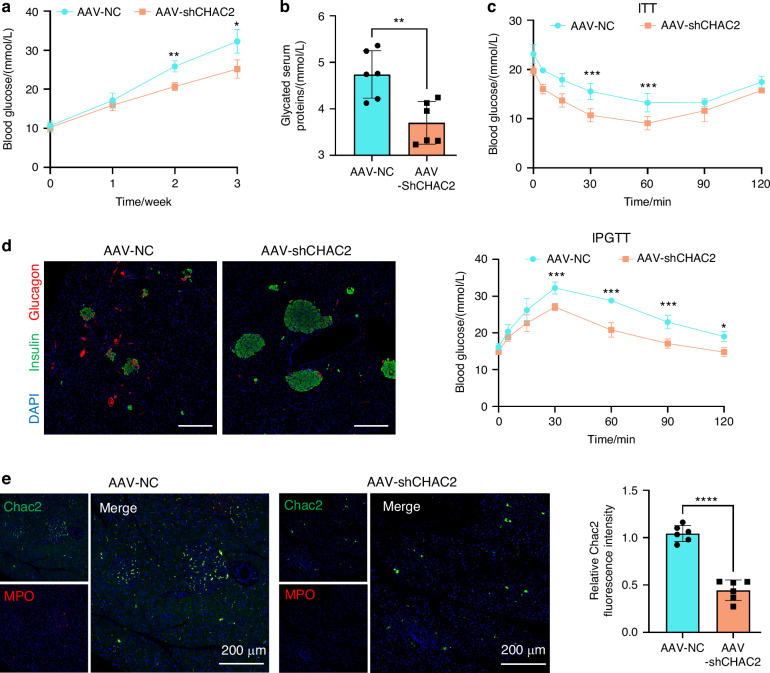
AAV-shChac2 delivery alleviates N1 neutrophil-mediated systemic inflammation. **a** Statistical curves of random blood glucose measurements in the AAV-NC and AAV-shCHAC2 groups. **b** Quantitative analysis of serum glycated protein content. **c** Statistical curves of blood glucose measurements during ITT and IPGTT. **d** Representative immunofluorescent staining of glucagon (green), insulin (red), and cell nuclei (DAPI, blue). Scale bar = 200 μm. **e** Representative immunofluorescent staining and of Chac2 (green), MPO (red), and cell nuclei (DAPI, blue) in pancreatic tissue sections and quantification of Chac2 fluorescence intensity. Scale bar = 200 μm. Data are presented as the mean ± SD. The *P* values were calculated using two-tailed Student’s t test; *P < 0.05, **P < 0.01, ***P < 0.001, ****P < 0.000 1, ns indicates no significant difference

To explore whether targeted intervention of Chac2 in neutrophils could alleviate the inflammatory comorbidity of periodontitis, we constructed AAV9 viruses targeting neutrophils to specifically knock down Chac2 (AAV-shCHAC2) and a control virus with no payload (AAV-NC), and administered them via intraosseous injection into a mouse model with periodontitis and diabetes comorbidity. FCM analysis confirmed the neutrophil-targeting specificity of the AAV construct, as the GFP signal was selectively enriched in BM neutrophils, compared to macrophages or other myeloid cells (Fig S9b). During three weeks of modeling, the random blood glucose and glycated serum protein levels of the AAV-shCHAC2 group were significantly lower than those of the control group (Fig. 8a, b), and islet dysfunction, as indicated by IPGTT, ITT and insulin/glucagon fluorescence staining of pancreatic tissue, was alleviated (Fig. 8c, d). Double immunofluorescence staining for detection of MPO/Chac2 and FCM analysis of Chac2 expression in pancreas demonstrated the effective targeting and knockdown of neutrophils by AAV treatment (Fig. 8e and Fig S9c). These results thus suggest that Chac2-regulated N1 polarization of BM neutrophils plays a key role in the pathological process by which periodontitis exacerbates diabetic comorbidity.

Together, these results functionally demonstrated that intracellular GSH elevation promotes neutrophil N1 polarization, which in turn contributes to the exacerbation of diabetic comorbidity in the context of periodontitis. Correspondingly, targeted knockdown of Chac2 in BM neutrophils can reverse N1 polarization and effectively alleviate the aggravating effects of local chronic inflammation on distal comorbidities. This IFN-I–CHAC2–GSH signaling axis thus represents a critical upstream regulatory pathway linking local chronic inflammation to systemic neutrophil activation and immune-metabolic dysfunction.

## Discussion

**Fig. 9:**
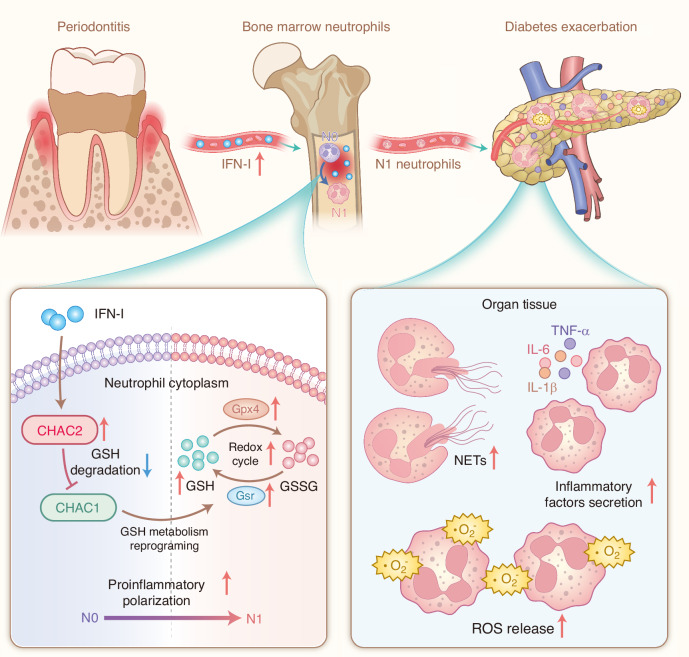
Schematic diagram illustrating CHAC2-mediated GSH metabolic reprogramming drives N1 polarization of BM neutrophils and exacerbates inflammatory comorbidities

There is emerging evidence that local chronic inflammation, such as periodontitis, is a key contributor to systemic immune dysregulation and multi-organ comorbidities.^48,49^ To delineate the role of BM neutrophils in this process, we employed integrated metabolomic and transcriptomic profiling and demonstrated that periodontitis triggers their polarization toward a pro-inflammatory N1 phenotype via GSH metabolic reprogramming. This reprogramming is driven by IFN-I signaling, which upregulates Chac2 expression at the transcriptional level. In contrast to its canonical role in GSH degradation, we found that in the context of inflammation, Chac2 upregulation led to a pronounced intracellular accumulation of GSH, which in turn enhanced neutrophil N1 effector functions. These insights thus broaden the conceptual framework of neutrophil immunometabolism and suggest that targeting the IFN-I–Chac2–GSH signaling axis may provide novel strategies for mitigating inflammation-driven comorbidities (Fig. 9).

Neutrophils are capable of undergoing N1 polarization across diverse pathological microenvironments,^27,50,51^ but their functional properties, metabolic basis, and regulatory mechanisms exhibit fundamental divergences and nuances that vary according to the disease condition and state. For instance, within the tumor microenvironment, N1-polarized neutrophils exert anti-tumor protective effects by generating ROS, NETs, and proinflammatory cytokines, which directly kill tumor cells and activate adaptive immunity.^28,52,53^ Over the past 5 years, a growing body of works have extended this concept to non-malignant inflammatory diseases (eg. ischemic stroke,^54–56^ myocardial infarction,^50,57–59^ rheumatoid arthritis^60,61^ and inflammatory bowel disease^62^) and has begun to consolidate a functional consensus. Across these contexts, N1-type neutrophils are consistently defined by a proinflammatory program that amplifies inflammatory cascades, disrupts tissue homeostasis, and drives multi-organ dysfunction.^63,64^ Accordingly, N1 neutrophils are typically characterized by a coordinated effector program that includes enhanced ROS generation, increased NETs formation, and elevated production of proinflammatory cytokines, together with transcriptional signatures enriched for chemokines, costimulatory and adhesion molecules.^7,65,66^ Our study revealed that this functional divergence originates from distinct metabolic reprogramming processes. For example, tumor-associated N1 polarization is primarily driven by local cytokines such as TGF-β and IFN-γ,^53^ and is often intertwined with hypoxia-induced glycolysis and the dynamics of N2 polarization,^67,68^ but lacks well-defined metabolic signatures. By comparison, in the BM microenvironment affected by peripheral inflammation, N1 polarization is orchestrated by systemic IFN-I signaling and is precisely regulated through a newly identified IFN-I–Chac2–GSH signaling axis.

This study specifically uncovers a non-canonical role of GSH metabolism within the BM microenvironment remodeled by peripheral inflammation. Traditionally, GSH has been regarded as a key antioxidant maintaining redox homeostasis, primarily functioning through the GSH redox cycle to scavenge ROS and preserve cell survival.^29,30^ However, in the periodontitis model, we observed a fundamental reprogramming of GSH metabolism in BM neutrophil, with IFN-I signaling markedly upregulating the degradation-regulatory protein Chac2, which can in turn repressed the catalytic activity of Chac1,^38–40,69^ resulting in substantial intracellular GSH accumulation. This accumulation was accompanied by enhanced expression of Gpx4 and Gsr, thereby strengthening redox cycling efficiency and providing essential metabolic support for sustained ROS generation and NETs formation. We demonstrated that Chac2-mediated GSH metabolic remodeling constitutes the core metabolic basis for the pro-inflammatory functions of N1-polarized neutrophils. By preventing collapse from oxidative stress while simultaneously fueling inflammatory effector activity, this pathway enables neutrophils to maintain persistent pro-inflammatory outputs. These findings thus challenge the conventional view of GSH metabolism as a passive redox buffer and instead establish it as an active driver of inflammatory processes.

From a mechanistic perspective, the rate of GSH consumption in neutrophils under inflammatory stress greatly exceeds its rate of synthesis,^70^ with Chac2-mediated expansion of the intracellular GSH pool emerging as a critical compensatory adaptation. This study not only uncovers the central role of Chac2 in immunometabolism reprogramming for the first time, but also provides a new perspective for understanding how periodontal inflammation alters neutrophil functional states to influence systemic disease progression. More importantly, we demonstrated that within the BM microenvironment remodeled by periodontitis, neutrophils undergo N1 polarization through an IFN-I–Chac2–GSH signaling axis, thereby exacerbating comorbidities such as diabetes. This finding thus underscores that periodontal inflammation is not merely an isolated oral condition but a potent driver of distal tissue damage and systemic comorbidity progression through immunometabolic reprogramming.

Therefore, controlling periodontal inflammation is not only critical for maintaining oral health but also essential for preventing systemic dissemination of inflammation and reducing the risks of comorbidities.^49,71,72^ Hence, targeting the Chac2–GSH signaling axis offers a promising strategy for precise intervention in neutrophil pathogenic activation, thereby providing a novel therapeutic avenue for mitigating periodontitis-associated systemic inflammatory responses. From the perspective of immunometabolism, this study further underscores the importance of periodontal disease management in maintaining systemic health, offering a theoretical foundation for clinical practice in which controlling local oral inflammation may slow the progression of systemic comorbidities.

### Limitations

Several limitations of this study should be acknowledged. First, while we identified IFN-I signaling as a key upstream inducer of Chac2 expression, the precise molecular details by which IFN-I regulates Chac2 transcription and activity require further investigation. In addition, although our mechanistic gain- and loss-of-function studies were supported by extensive in vivo validation, part of the functional characterization was performed using HL60-derived neutrophil cells. While this model enables efficient genetic manipulation and has been widely used to in neutrophil researches, it may not completely recapitulate the metabolic state and signaling complexity of primary BM neutrophils in vivo. Moreover, the downstream mechanisms by which GSH metabolic remodeling supports neutrophil effector functions might through multiple processes such as reinforcing redox cycling capacity, stabilizing ROS-generating systems during respiratory burst, and preserving neutrophil viability under oxidative stress. While additional redox-sensitive signaling intermediates (e.g., kinases, transcription factors, or inflammasome-related pathways) will need to be delineated in future studies.

Second, our in vivo comorbidities studies were conducted using the *db/db* mouse model, which is widely used and clinically relevant. However, this model may not fully reflect the complexity of diabetes in patients. Future studies employing complementary models and clinical samples will be necessary to further substantiate the translational relevance of our findings.

Finally, while our results suggest promising therapeutic potential for targeting Chac2, innovative drug delivery strategies to achieve non-invasive and precise modulation of the neutrophil IFN-I–Chac2–GSH signaling axis in clinical settings remain to be developed. Addressing these questions will thus refine our understanding of the IFN-I–Chac2–GSH signaling axis and strengthen its potential as a therapeutic target in inflammation-driven comorbidities.

## Conclusion

Within the BM microenvironment remodeled by periodontitis, neutrophils acquire pro-inflammatory functions by reprogramming their metabolic networks, particularly GSH metabolism. In this context, GSH metabolism is no longer merely a biosynthetic substrate but serves as a central regulatory switch that directly governs neutrophil functional states. The large pool of neutrophils generated through hematopoietic skewing after being reprogrammed by GSH metabolism plays a pivotal role in propagating systemic inflammation. These findings thus provide mechanistic insights into how local periodontal inflammation exacerbates systemic comorbidities and underscore the critical importance of managing local inflammatory diseases.

## Materials and methods

### Mice

C57BL/6 wild-type mice were purchased from Beijing Vital River Laboratory Animal Technology Co., Ltd. (Beijing, China). *Ifnar1*^*-/-*^ mice were obtained from Cyagen Bioscience Inc. (Guangzhou, China). The *db/db* mice were purchased from GemPharmatech Co., Ltd (Jiangsu, China). Six-week-old male mice were used for all experiments. Mice were housed in specific pathogen-free conditions with a 12: 12 h light/dark cycle with a temperature of 24 °C ± 0.5 °C and a relative humidity of 40%-70%. Food and water were provided *ad libitum* during the experimental period. Animal experiments were approved by the Institutional Animal Care and Use Committee of Peking University Health Science Center (approved number: DLASBD0673).

### Ligature-induced periodontitis

To investigate the impact of experimental periodontitis on BM neutrophils, Ligature induced periodontitis was performed in mice. In brief, bilateral maxillary and mandibular second molars were both tied with 5-0 silk ligatures for 14 days to induce periodontitis. Control mice did not undergo ligature placement on their teeth.

### Cell preparations and sample collection

For BM neutrophil isolation, femurs and tibiae from C57BL/6 mice were flushed with ice-cold RPMI 1640 medium. The cells were then passed through a 70 µm nylon mesh to obtain a single-cell suspension for subsequent flow cytometric analysis and FACS cell sorting. To collect BM extracellular fluid, femurs and tibiae were flushed with 1 mL of ice-cold PBS, and the supernatant was collected after centrifugation at 500 × *g* for 5 min at 4 °C. Whole-blood samples were obtained through retrobulbar bleeding.

### Flow cytometry and sorting

Flow cytometric analysis was performed using an LSRFortessa cytometer (BD Biosciences), cell sorting was performed using a FACSAria II sorter (BD Biosciences), and all data were analyzed with FlowJo software (Tree Star Inc.). For cell surface phenotyping, anti-CD11b (clone M1/70), anti-Ly6G (clone 1A8), anti-CD66a (clone MAb-CC1), and anti-F4/80 (clone W20065D) antibodies were used. Gating strategies for BM and pancreatic samples were defined as follows: neutrophils, CD11b⁺Ly6G⁺; monocytes, CD11b⁺Ly6G⁻F4/80⁺; and other myeloid cells, CD11b⁺Ly6G⁻F4/80⁻. Intracellular GSH levels were assessed using ThiolTracker Violet (T10095, Thermo Fisher Scientific), and ROS levels were measured using DCFDA (HY-D0940, MedChemExpress).

To preserve neutrophil viability and functional status, the entire procedure from mouse sample collection to completion of flow cytometric sorting was controlled within 6 h. All procedures during sample staining and washing were performed as gently as possible to minimize mechanical stress. Sorted cells were collected into buffer containing 2% FBS. After sorting, neutrophils were centrifuged at 500 × g at 4 °C and resuspended in RPMI-1640 medium with 10% FBS for subsequent culture, stimulation and functional assays. Pilot experiments confirmed a post-sorting cell viability of greater than 75% by trypan blue straining.

### Cytokine assay

The sorted BM neutrophils were cultured in RPMI 1640 medium (ThermoFisher) supplemented with 10% (v/v) FBS for 30 min. The neutrophils were then seeded into 12-well plates (1 × 10^6^ cells per well) and stimulated with 150 ng/mL E. coli O111 : B4 LPS (InVivogen) for 17 h. Subsequently, the cell culture supernatants were collected for cytokine analysis. Cytokine levels were assessed using a Proteome Profiler Mouse Cytokine Array Kit (R&D Systems) according to the manufacturer’s instructions. In addition, the concentrations of IL-1β, IL-6, and TNF-α were specifically quantified using mouse ELISA kits (Absin). The Mouse IFN-beta Quantikine ELISA Kit (R&D Systems) and Mouse IFN-alpha All Subtype Quantikine ELISA Kit (R&D Systems) were utilized to determine the concentrations of IFNα and IFNβ in the BM extracellular fluid, respectively, following the manufacturer’s instructions.

### Histology and immunostaining

Mouse pancreases were fixed with 4% (w/v) paraformaldehyde, dehydrated in a graded series of alcohol, paraffin-embedded, and tissue sectioned to 5 µm for histological evaluation. For immunofluorescence staining of pancreas, the slides were deparaffinized and subjected to antigen retrieval, then permeabilized, blocked, and incubated with various antibodies, including anti-Glucagon (ab92517, Abcam), anti-Insulin (ab181547, Abcam), anti-Chac2 (16304-1-AP, Proteintech), and anti-MPO (AF3667, R&D systems). The images were then scanned with ZEISS AXIOSCAN 7 and analyzed with the ImageJ software.

### Immunofluorescence cell staining

The cells were gently rinsed with PBS and fixed in 4% (w/v) paraformaldehyde for 15 min. Permeabilization was performed using 0.5% Triton X-100 (Sigma) for 5 min at room temperature. The cells were then blocked with 3% BSA (Solarbio) for 30 minutes before overnight incubation at 4 °C with primary antibodies, including anti-MPO (AF3667, R&D Systems) and anti-CitH3 (ab5103, Abcam) antibodies. Following primary antibody incubation, cells were treated with goat anti-rabbit IgG H&L Alexa Fluor 488 (ab150077, Abcam), donkey anti-goat IgG H&L Alexa Fluor 647 (ab150131, Abcam) and DAPI (Invitrogen) for 1 hour in the dark at room temperature. The images were acquired using a TCS-SP8 STED 3X microscope and analyzed with Fiji software (v 2.0.0).

### Adoptive transfer of neutrophils

BM neutrophils from donor mice (Con or Lig) were sorted by FACS and subsequently used for the indicated stimulation by GDE (5 mM) and L-BSO (100 μmol/L) for 24 h. Each recipient *db/db* mouse received tail vein injections of 5 × 10^6^ neutrophils every 5 days. Three weeks after adoptive transfer, the pancreatic tissues and whole blood were collected for tissue section staining and detection of glycated serum proteins to assess the impact of polarized N1 neutrophils on diabetes in recipient mice.

### RT-qPCR

Total RNA was isolated from the BM neutrophils or HL60 cells using SteadyPure Quick RNA Extraction Kit (AG21023, Accurate Biotechnology) according to the manufacturer’s instructions. cDNA was synthesized using the Evo M-MLV RT Premix (AG11706, Accurate Biotechnology), according to the manufacturer’s instructions. RT-qPCR was conducted using the Hieff UNICON^®^ Universal Blue qPCR SYBR Green Master Mix (11184ES08, Yeasen) together with specific primers, and analyzed by real-time fluorescence quantitative PCR instrument (QuantStudio 3, ThermoFisher). The gene expression levels were normalized to *Gapdh* mRNA levels. The primer sequences are listed in Supplementary Table 1.

### PCR array

The pro-inflammatory N1 polarization of neutrophils was assessed using the Inflammatory Cytokines & Receptors PCR Array (WC-MRNA0266, WcGene Biotech), which analyzed a total of 90 genes. GAPDH, 18S, β-Actin, and HPRT1 were utilized as internal control genes. Each plate included two blank wells as negative controls.

### Chac2 knockdown and overexpression

To knock down the expression of *CHAC2* in HL60 neutrophils, we utilized specific shRNA sequences as follows: 5’-GCTACAGAACCACAACAGTCA-3’. A non-targeting shRNA sequence was used as a control. The HL60 cells were transfected with CHAC-targeting shRNA lentiviral particles (Sh-CHAC2) or control shRNA lentiviral particles (Sh-NC) using Lipofectamine 3000 (Invitrogen) according to the manufacturer’s instructions. After 48 hours of transfection, the cells were cultured in complete medium supplemented with 2 µg/mL puromycin for 72 hours to select the successfully transfected cells. The efficiency of gene knockdown was confirmed by RT-PCR and WB. These selected cells were then used for downstream experimental analyses to ensure the purity and reliability of the results. The full-length *CHAC2* coding sequence was cloned into a lentiviral expression vector (pRRLSIN-cPPT-SFFV-MCS-3FLAG-E2A-EGFP-SV40-puro). Lentivirus was produced in HEK293T cells by co-transfection with packaging plasmids and subsequently used to transfect HL-60 cells. The transduced cells were then selected with puromycin (1 μg/mL) for 5–7 days to establish stable lines. Overexpression efficiency was confirmed by qRT-PCR and Western blot analysis. The empty vector-transduced HL-60 cells served as controls. HL60 cells were differentiated into HL60-neutrophils by treatment with 1.25% (v/v) DMSO for 5 days and were then used for subsequent functional assays.

### AAV production for Chac2 silencing

To construct AAV vectors for the specific silencing of Chac2 in mouse BM neutrophils, we first selected target sequences for mouse Chac2 mRNA using the application program from Dharmacon siDESIGN center (http://www.dharmacon.com). The specific shRNA sequence 5’-CTACAGAACTACGACAGTCAT-3’ was designed. A non-specific control shRNA sequence (shNC) was also designed to serve as the negative control, ensuring no significant sequence similarity to any known mouse genes. These sequences were cloned into an AAV vector backbone in which a Ly6G promoter–driven cassette was engineered to co-express GFP, allowing visualization of transduced Ly6G⁺ neutrophils in vivo, while a U6 promoter was used to drive shRNA expression. For virus production, the recombinant plasmids were co-transfected with helper plasmids into HEK293 cells using Lipofectamine 3000 transfection reagent (L3000150, Invitrogen). The helper plasmids provided necessary AAV rep and cap genes, as well as adenoviral helper functions. The virus-containing medium was harvested at 72 hours post-transfection, and the virus particles were purified through gradient ultracentrifugation. The titer of the AAV particles was determined by the AAV Quantitation Titer Kit (Cell Biolabs, San Diego, CA, USA).

### AAV-shCHAC2 intraosseous injection of *db/db* mice with periodontitis

Following 14 days of silk ligation-induced periodontitis, *db/db* mice were anesthetized with isoflurane, and both knees were flexed with support behind each knee. Hair was shaved around the joint area, and 70% (v/v) alcohol and iodine were used to clean the area. A 1 ml syringe with a 25 (5/8 length) gauge needle was inserted into the intrafemoral space by gentle twisting and application of pressure between the condyles at the top of the femur between the tibia and femur joint. The 25 (5/8 length) gauge needle and cap were left in place, while the 1 mL syringe was gently removed. A (25 μL 1702 RN) Hamilton syringe with a 32 G needle (7803-04, 32 Gauge RN 2” point size 4, referred to as bone marrow needles) was inserted into the plastic cap opening and threaded through the needle opening of the 25 (5/8 length) gauge needle. The 32 G bone marrow needle was marked at 3.5 cm from the tip to indicate the length at which to discontinue insertion. Five microliters of solution (AAV-GFP (4.5 × 10^13^ CFU/mL, AAV-shCHAC2 (1 × 10^13^ TU/mL) was slowly injected by free hand into the shaft of the femur using the 25 μL Hamilton syringe and slowly removed to limit backflow. The 25 (5/8 length) gauge needle was then gently removed, and mice were monitored and allowed to recover.

### Pancreas dissociation and flow cytometric analysis of infiltrating neutrophils

Mouse pancreas were harvested and immediately processed for single-cell preparation. Pancreatic tissues were minced into small fragments and enzymatically digested in RPMI-1640 containing 0.5 mg/mL collagenase P and 1 mg/mL DNase I at 37 °C with gentle agitation. Digestion was stopped by adding ice-cold staining buffer (PBS supplemented with 2% FBS), and the resulting suspension was filtered through a 70-μm cell strainer to remove undigested debris. After washing, cells were resuspended in staining buffer and analyzed on a flow cytometer. For neutrophil quantification, single-cell suspensions were stained with antibodies for 30 min at 4 °C in the dark, and then flow cytometry was performed.

For tracking of adoptively transferred neutrophils, BM neutrophils were isolated from donor mice and labeled ex vivo with CellTrace CFSE (ThermoFisher) in PBS at 37 °C, and then transferred into recipient mice via tail vein injection in 1 h. At 12 h after transfer, pancreas were collected and dissociated into single-cell suspensions as described above, and directly analyzed by flow cytometry to detect CFSE fluorescence in pancreatic cell suspensions.

### RNA sequencing analysis

Previously published RNA sequencing data were retrieved as raw files from the GEO database (GSE236477). These files were processed and transformed into Seurat-compatible objects for further analysis. All downstream bioinformatic analyses were performed using Omicsmart, a dynamic, real-time interactive online platform for data analysis (http://www.omicsmart.com).

### MESA enrichment analysis of metabolic pathways

Metabolite Set Enrichment Analysis (MSEA) was conducted using the MetaboAnalyst software (v6.0). The pathway-associated metabolite set was used as the metabolite library, with all compounds included. Pathways showing a Holm-adjusted *P* < 0.05 were considered statistically significant in pairwise comparisons across different time points.

### Glucose and insulin tolerance tests

The blood glucose levels of mice were assessed using an electronic dehydrogenase blood glucose meter (Yuwell 921), determined from tail vein samples, with RBG measured at 3 weeks after ligature. Both the IPGTT and the ITT were conducted. These tests were performed once, at 30 days post-intervention, and were not carried out before the intervention. For IPGTT, the mice fasted for 14 h and received a 20% glucose injection at 2 g/kg, with glucose levels being measured at 0, 30, 60, and 120 min post-injection for IPGTT calculation. In the ITT, mice were fasted for 6 h and were then injected with insulin at 0.5 IU/kg, with plasma glucose being measured at the same time point as IPGTT.

### Statistics

The animals were randomly assigned to treatment or control groups. All experiments were performed with independent biological replicates. The exact number of independent replicates (*n*) is indicated in the corresponding Fig. legends. The two-tailed Student’s *t*-test was utilized to compare data between the two independent groups. For data involving three or more groups with a single variable, the one-way ANOVA test was employed, followed by Tukey’s tests for multiple comparisons. Statistical analysis was conducted using the GraphPad Prism 9.0.2 software. All data are presented as mean ± SD, and statistical significance was defined as *P* < 0.05.

## Supplementary information

Supplemental Material

## Data Availability

All original data used for this study are available from the corresponding authors upon reasonable request.
