# Supplementary material for: CHAC2-mediated glutathione metabolic reprogramming drives N1 polarization of bone marrow neutrophils and exacerbates inflammatory comorbidities
Source: Int J Oral Sci. 2026 Jul 9;18:49. doi: 10.1038/s41368-026-00451-6 (PMC13351030; doi:10.1038/s41368-026-00451-6)
Supplement: Supplementary file 1 — Supplemental Material [file 41368_2026_451_MOESM1_ESM.docx]

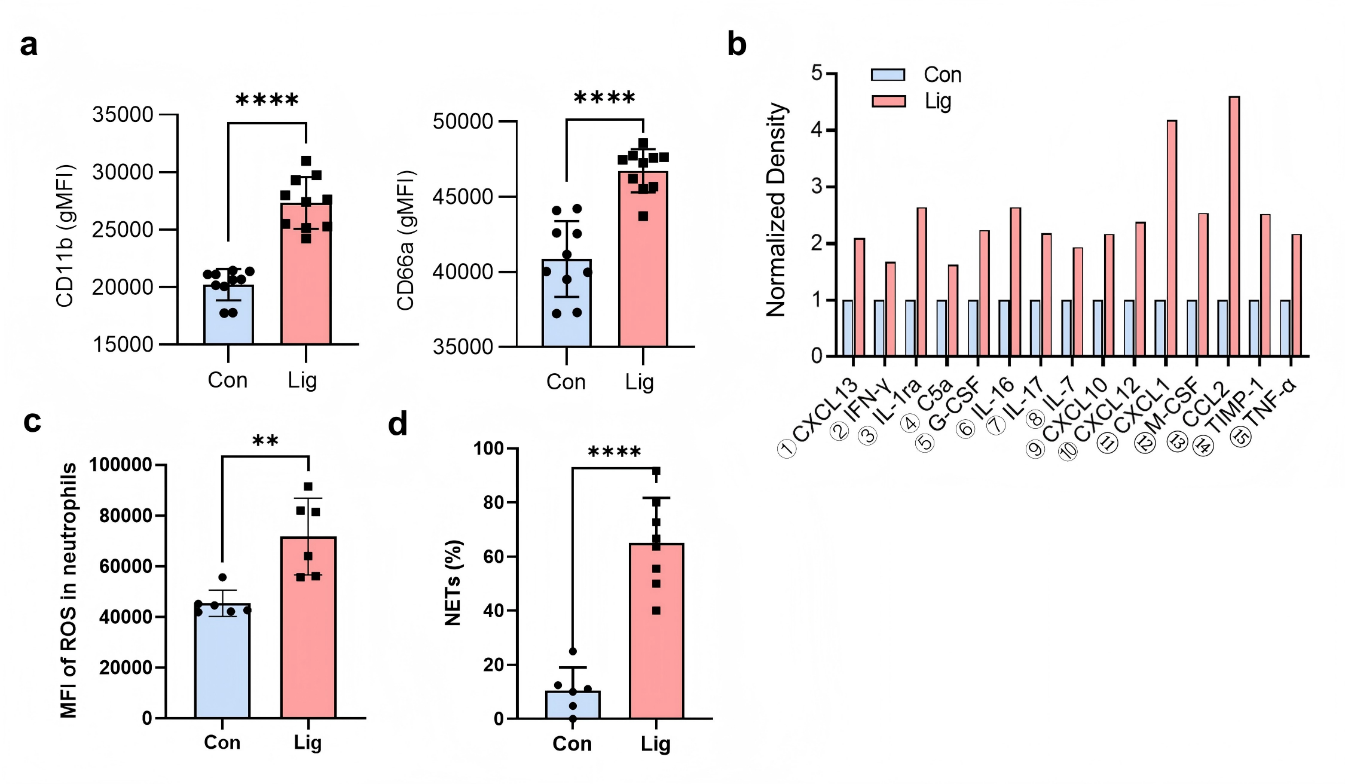


**Fig. S1 Periodontitis promotes a pro-inflammatory phenotype and functional activation of bone marrow neutrophils**

**(a)** Quantification of flow cytometric analysis of the expression of CD11b and CD66a in neutrophils obtained from the BM. **(b)** Relative quantitative statistics of cytokine antibody array. **(c)** Mean fluorescence intensity of ROS production in neutrophils. **(d)** Percentage of NETs area of neutrophils normalized to an MPO-citH3 positive signal. All data are presented as the mean ± SD from at least three independent replicate experiments. *P* values were calculated using two-tailed Student's t test; **P* <0.05, ***P* <0.01, ****P* <0.001, *****P* <0.0001, ns indicates no significant difference.


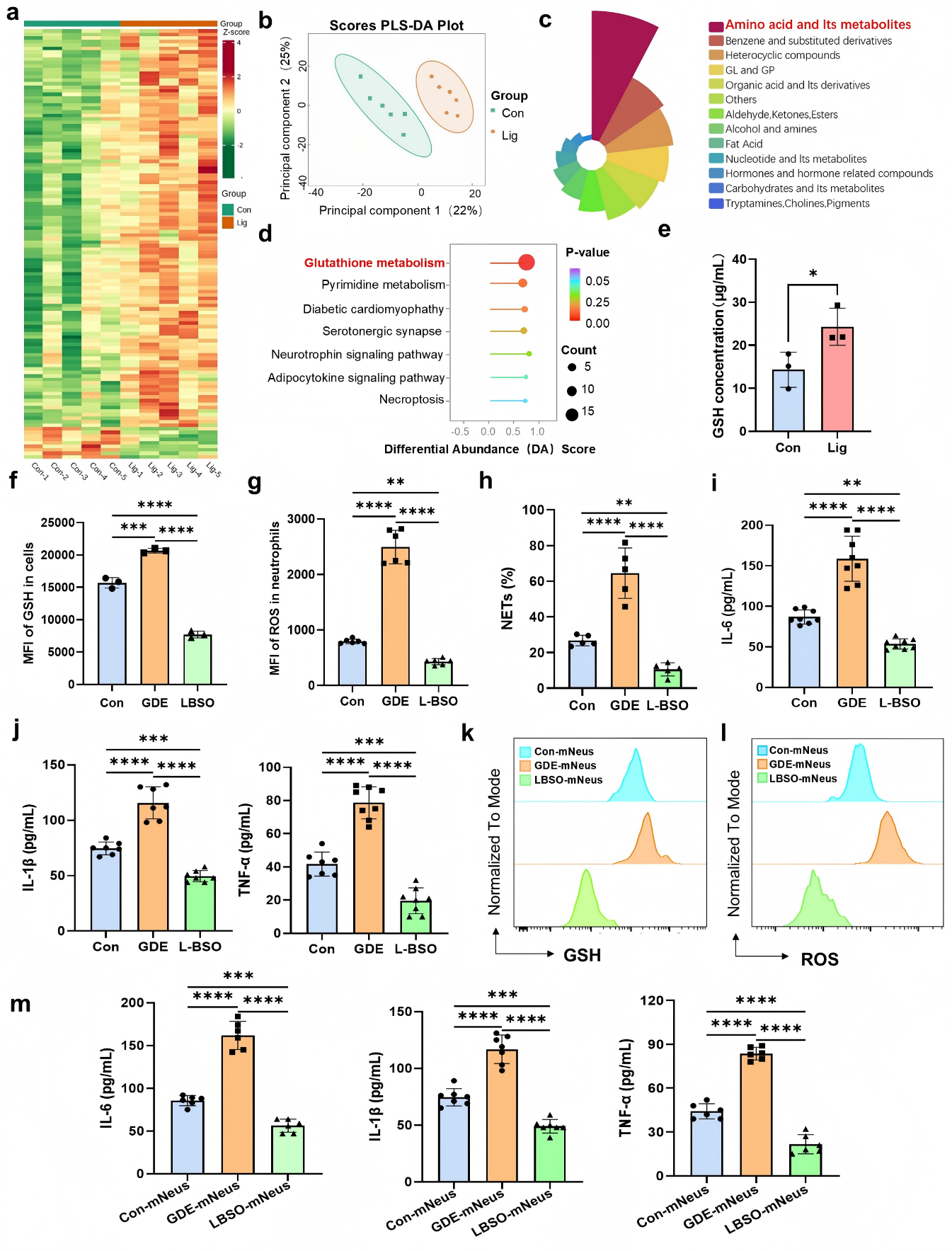


**Fig. S2 Modulation of glutathione levels drives N1 pro-inflammatory activation in bone marrow neutrophils**

**(a)** Heat map showing normalized metabolite levels in BM neutrophils from the Con and Lig groups. Quantification of total GSH concentrations in BM neutrophils from the Con and Lig mice, measured by ELISA. **(b)** PLS-DA of normalized metabolite data from the Con group and Lig group (n = 6 per group). **(c)** Nightingale rose chart of classification of differential metabolites. **(d)** MSEA enrichment analysis for metabolites differentially upregulated in the Lig group versus the Con group. **(e)** Quantification of GSH concentration in Con, GDE and L-BSO neutrophils. **(f)** Corresponding mean fluorescence intensity of GSH in Con, GDE and L-BSO neutrophils. **(g)** Corresponding mean fluorescence intensity of ROS production in neutrophils. **(h)** Percentage of NETs area in the Con, GDE and L-BSO HL60 neutrophils normalized to an MPO-citH3 positive signal. **(i)** Quantitative statistical analysis of reactive cytokines IL-6 produced by neutrophils. **(j)** Quantitative statistical analysis of reactive cytokines IL-1β, and TNF-α produced by neutrophils. **(k)** Overlay flow cytometry histograms and quantification of intracellular GSH levels in primary BM neutrophils under Con, GDE, or L-BSO treatment. **(l)** Overlay flow cytometry histograms and quantification of intracellular ROS levels in primary BM neutrophils under Con, GDE, or L-BSO treatment. **(m)** Quantification of proinflammatory cytokine production by primary BM neutrophils under Con, GDE, or L-BSO treatment. Data are presented as the mean ± SD. *P* values were calculated using two-tailed Student's t test (e) and one-way ANOVA associated with Tukey’s multiple comparisons test (f-m); **P* <0.05, ***P* <0.01, ****P* <0.001, *****P* <0.0001.


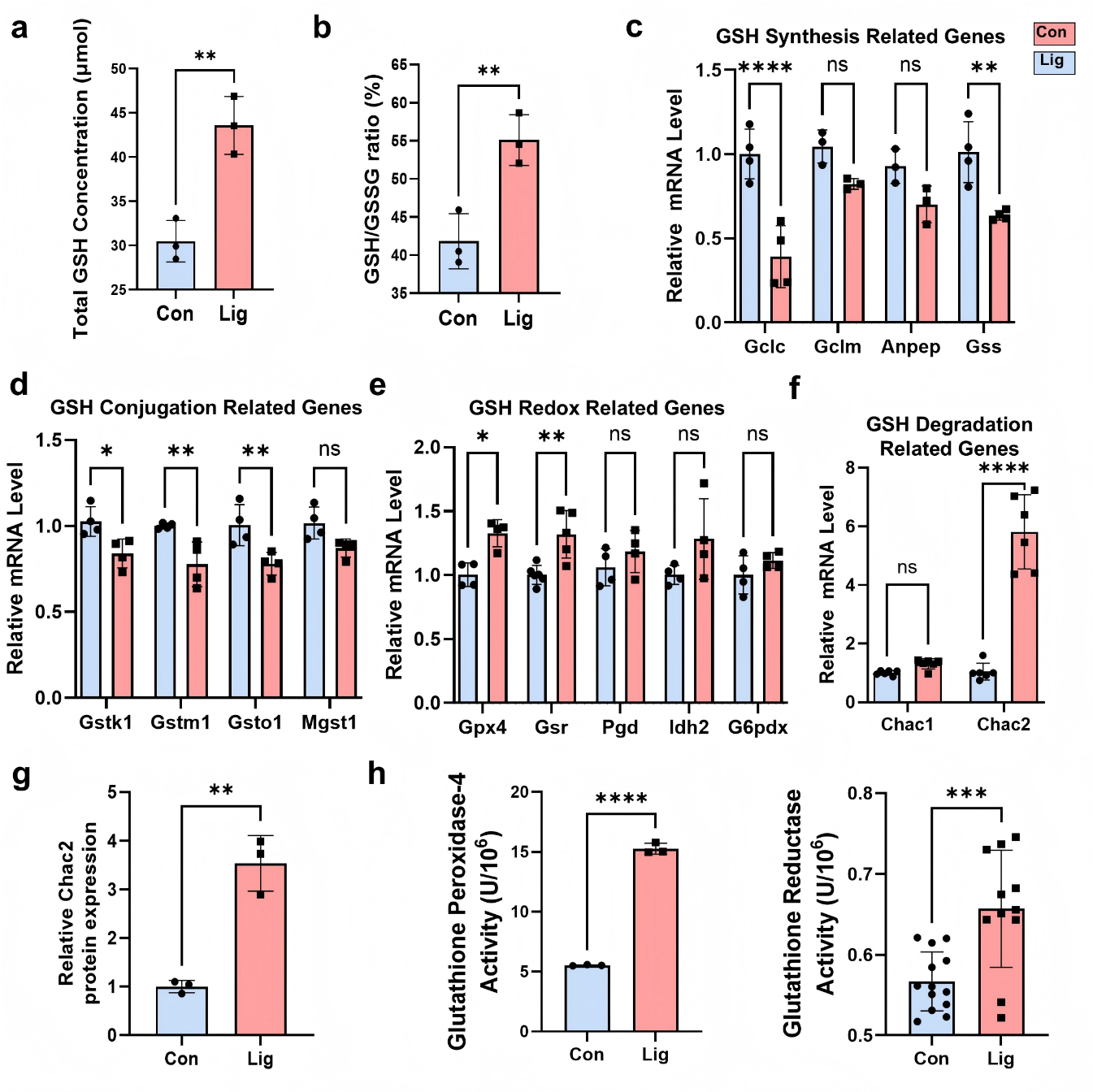


**Fig. S3 Glutathione metabolism reprograming in BM neutrophils from periodontitis mice**

**(a)** Quantitative statistics of the total GSH concentration in BM neutrophils. **(b)** Quantitative statistics of the GSH/GSSG ratio in BM neutrophils. **(c)** Quantitative statistics of the relative mRNA expression levels of GSH synthesis processes*.* **(d)** Quantitative statistics of the relative mRNA expression levels of GSH conjugation processes*.* **(e)** Quantitative statistics of the relative mRNA expression levels of GSH redox processes*.* **(f)** Quantitative statistics of the relative mRNA expression levels of GSH degradation processes*.* **(g)** Quantitative analyses of Chac2 protein expression levels in BM neutrophils from Con versus Lig mice. **(h)**The enzymatic activities of glutathione peroxidase-4 and glutathione reductase in Con and Lig BM neutrophils. All data are presented as the mean ± SD from at least three independent experiments. *P* values were calculated using two-tailed Student's t test (a, b, g, h) and one-way ANOVA followed by Tukey’s multiple comparisons test (c-e); **P* <0.05, ***P* <0.01, ****P* <0.001, *****P* <0.0001, ns indicates no significant difference.


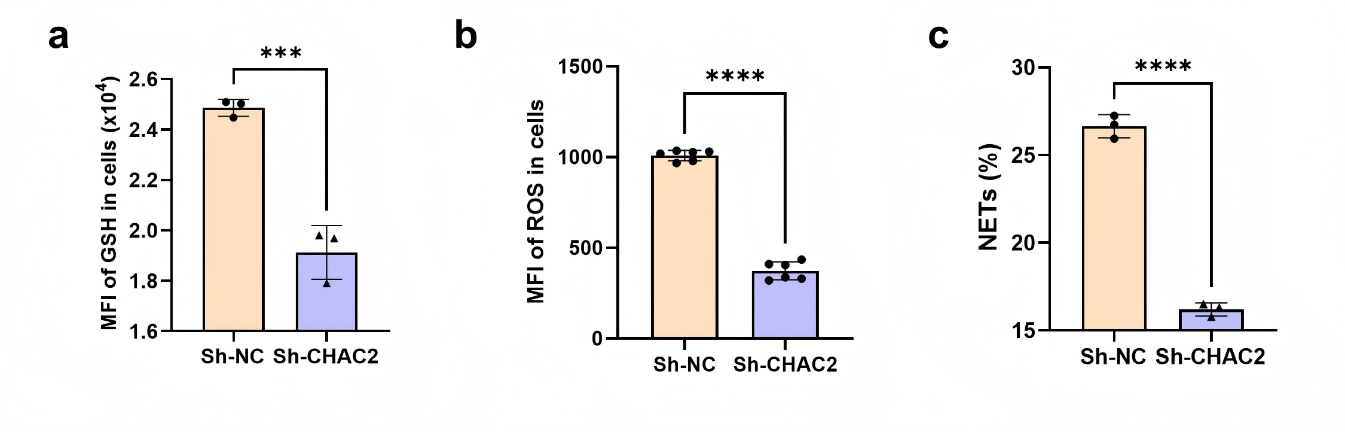


**Fig. S4 Knockdown of Chac2 reduces intracellular GSH levels, suppresses ROS production, and decreases NETs formation in neutrophils**

**(a)** Corresponding mean fluorescence intensity of GSH concentration in Sh-NC and Sh-Chac2 neutrophils. **(b)** Corresponding mean fluorescence intensity of ROS production in Sh-NC and Sh-Chac2 neutrophils. **(c)** Percentage of NETs area in the Sh-NC and Sh-Chac2 neutrophils normalized to an MPO-citH3 positive signal. All data are presented as the mean ± SD from at least three independent experiments. *P* values were calculated using two-tailed Student's t test; **P* <0.05, ***P* <0.01, ****P* <0.001, *****P* <0.0001, ns indicates no significant difference.


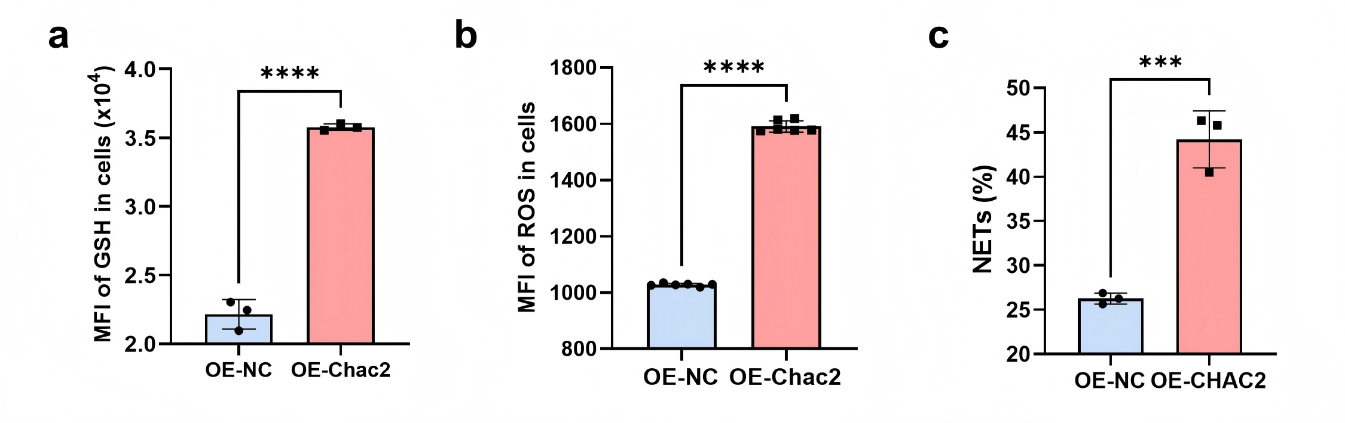


**Fig. S5 Chac2 overexpression increases intracellular GSH levels, enhances ROS production, and promotes NETs formation in neutrophils**

**(a)** Corresponding mean fluorescence intensity of GSH concentration in OE-NC and OE-Chac2 neutrophils. **(b)** Corresponding mean fluorescence intensity of ROS production in OE-NC and OE-Chac2 neutrophils. **(c)** Percentage of NETs area in the OE-NC and OE-Chac2 neutrophils normalized to an MPO-citH3 positive signal. All data are presented as the mean ± SD from at least three independent experiments. *P* values were calculated using two-tailed Student's t test; **P* <0.05, ***P* <0.01, ****P* <0.001, *****P* <0.0001, ns indicates no significant difference.


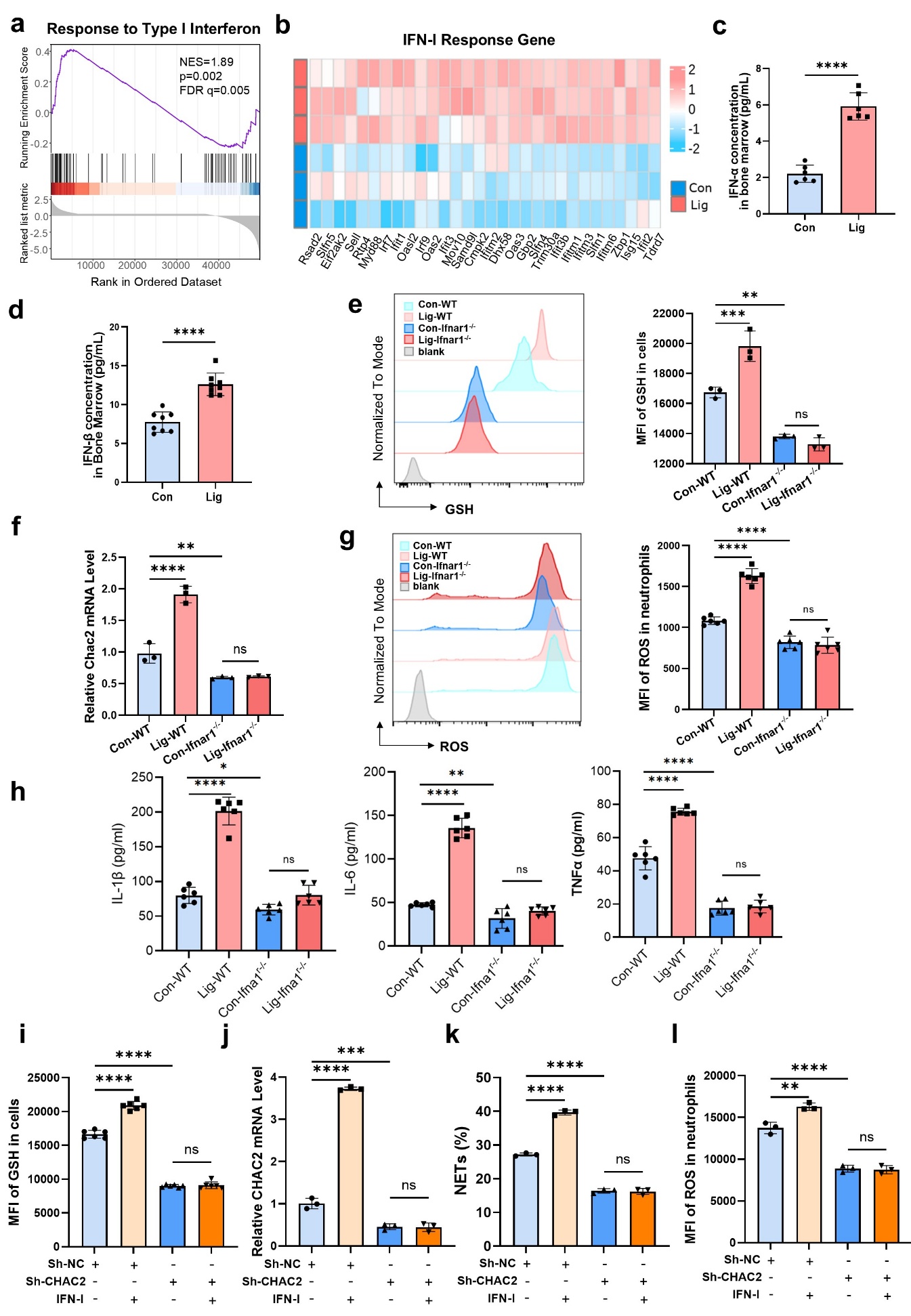


**Fig. S6 The IFN-I-Chac2 axis drives glutathione metabolic reprogramming and pro-inflammatory activation of neutrophils in periodontitis**

**(a)** GSEA analysis of upregulated genes expressed in the RNA-seq data of neutrophils. **(b)** Heatmap of the expression of IFN-I response-related gene sets in the RNA-seq data. **(c)** Quantification of IFN-α levels in the BM supernatant of Con and Lig mice. **(d)** Quantification of IFN-β levels in the BM supernatant of Con and Lig mice. **(e)** Overlay histogram and corresponding mean FCM fluorescence intensity of GSH concentration in Con-WT, Lig-WT, Con-Ifnar1^-/-^ and Lig-Ifnar1^-/-^ BM neutrophils. **(f)** Quantitative analysis of the relative Chac2 mRNA expression levels in Con-WT, Lig-WT, Con-Ifnar1^-/-^ and Lig-Ifnar1^-/-^ BM neutrophils. **(g)** Overlay histogram and corresponding mean fluorescence intensity of ROS production in neutrophils. **(h)** Quantitative statistical analysis of reactive cytokines IL-6, IL-1β, and TNF-α produced by neutrophils. **(i)** Corresponding mean FCM fluorescence intensity of GSH concentration in Sh-NC and Sh-CHAC2 neutrophils of the blank or IFN-I treatment groups. **(j)** Statistical chart of relative changes in CHAC2 mRNA expression levels. **(k)** Percentage of NETs area in the Sh-NC and Sh-Chac2 neutrophils treated with or without IFN-I normalized to an MPO-citH3 positive signal. **(l)** Quantification of ROS production in Sh-NC and Sh-CHAC2 neutrophils of the blank or IFN-I treatment groups. Data are presented as the mean ± SD from at least three independent experiments. *P* values were calculated using two-tailed Student's t test (c, d) and one-way ANOVA followed by Tukey’s multiple comparisons test (e-l); **P* <0.05, ***P* <0.01, ****P* <0.001, *****P* <0.0001, ns indicates no significant difference.


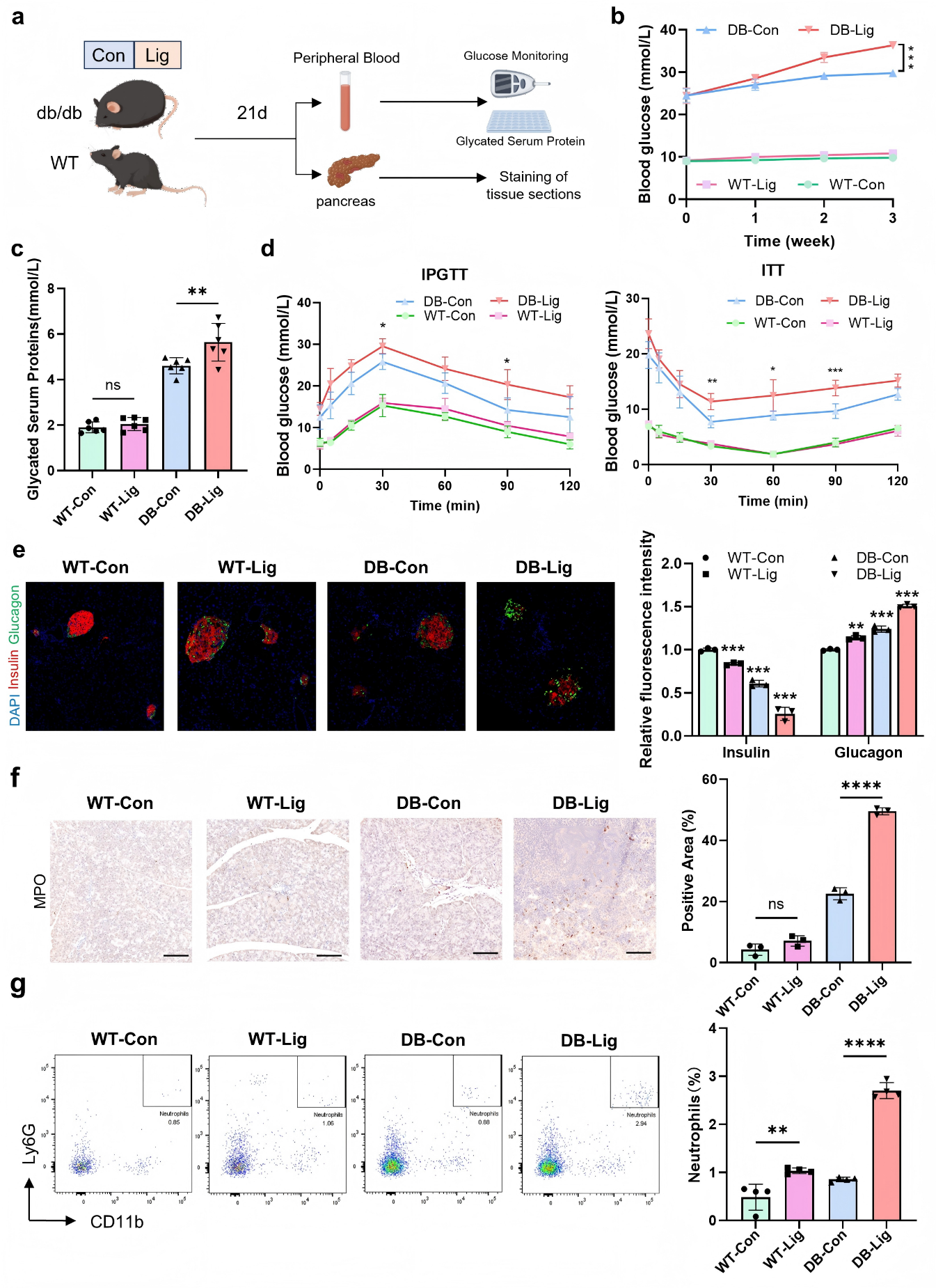


**Fig. S7 Periodontitis aggravates pancreatic dysfunction and neutrophil infiltration in a diabetic mouse model**

**(a)** Schematic illustration of the experimental design for detecting blood glucose and pancreatic tissue damage after implementing periodontitis ligation-induced comorbidity models or control groups in wild-type mice and *db/db* mice. **(b)** Statistical curves of random blood glucose measurements in the WT-Con, WT-Lig, DB-Con and DB-Lig groups. **(c)** Quantitative analysis of serum glycated protein content. **(d)** Statistical curves of blood glucose measurements during IPGTT and ITT. **(e)** Representative immunofluorescent staining of glucagon (green), insulin (red), and cell nuclei (DAPI, blue). **(f)** Representative immunohistochemical staining of MPO in pancreas tissue sections. Scale bar = 100 μm. **(g)** Representative flow cytometry dot plots showing the percentage and the statistic frequencies of pancreatic neutrophils (CD45^+^CD11b⁺Ly6G⁺) in WT-Con, WT-Lig, DB-Con and DB-Lig mice. Data are presented as the mean ± SD. *P* values were calculated using one-way ANOVA followed by Tukey’s multiple comparisons test;**P* <0.05, ***P* <0.01, ****P* <0.001, *****P* <0.0001, ns indicates no significant difference.


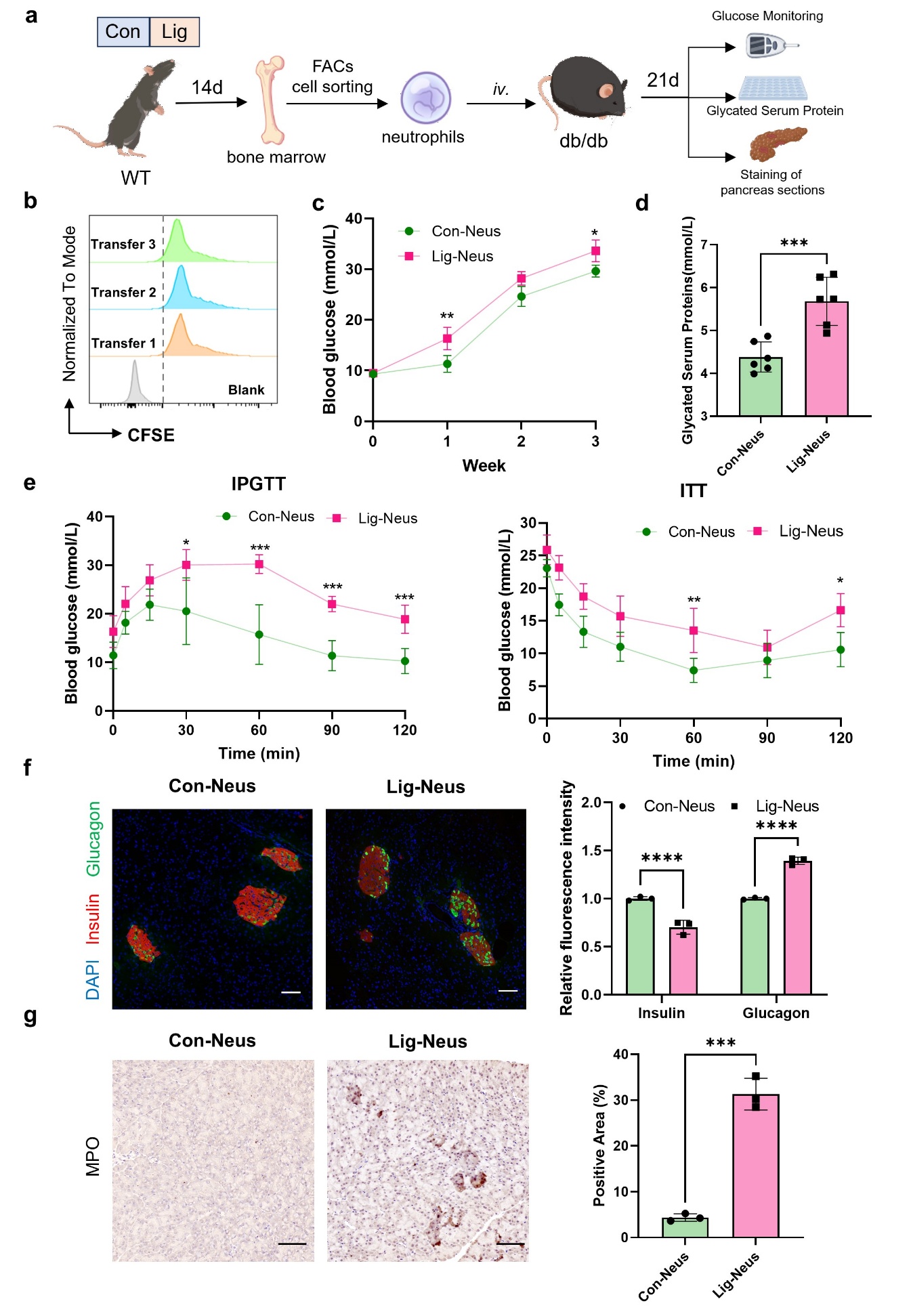


**Fig. S8 Adoptive transfer of periodontitis-primed bone marrow neutrophils promotes pancreatic inflammation and injury in *db/db* diabetic recipient mice**

**(a)** Schematic illustration of the experimental design for the transfer BM neutrophils from Con or Lig wild-type mice to *db/db* mice. **(b)** Representative FCM histograms of CFSE fluorescence in single-cell suspensions from mice pancreata 12 h after adoptive transfer of CFSE-labeled bone marrow neutrophils (Transfer 1-3) compared with non-transferred control mice (Blank). **(c)** Statistical curves of random blood glucose measurements in the Con-Neus and Lig-Neus groups. **(d)** Quantitative analysis of serum glycated protein content. **(e)** Statistical curves of blood glucose measurements during IPGTT and ITT. **(f)** Representative immunofluorescent staining of glucagon (green), insulin (red), and cell nuclei (DAPI, blue), and **(g)** immunohistochemical staining of MPO in pancreas tissue sections. Scale bar = 100 μm. Data are presented as the mean ± SD. *P* values were calculated using two-tailed Student's t test (c-e, g) and one-way ANOVA followed by Tukey’s multiple comparisons test (f); **P* <0.05, ***P* <0.01, ****P* <0.001, *****P* <0.0001, ns indicates no significant difference.


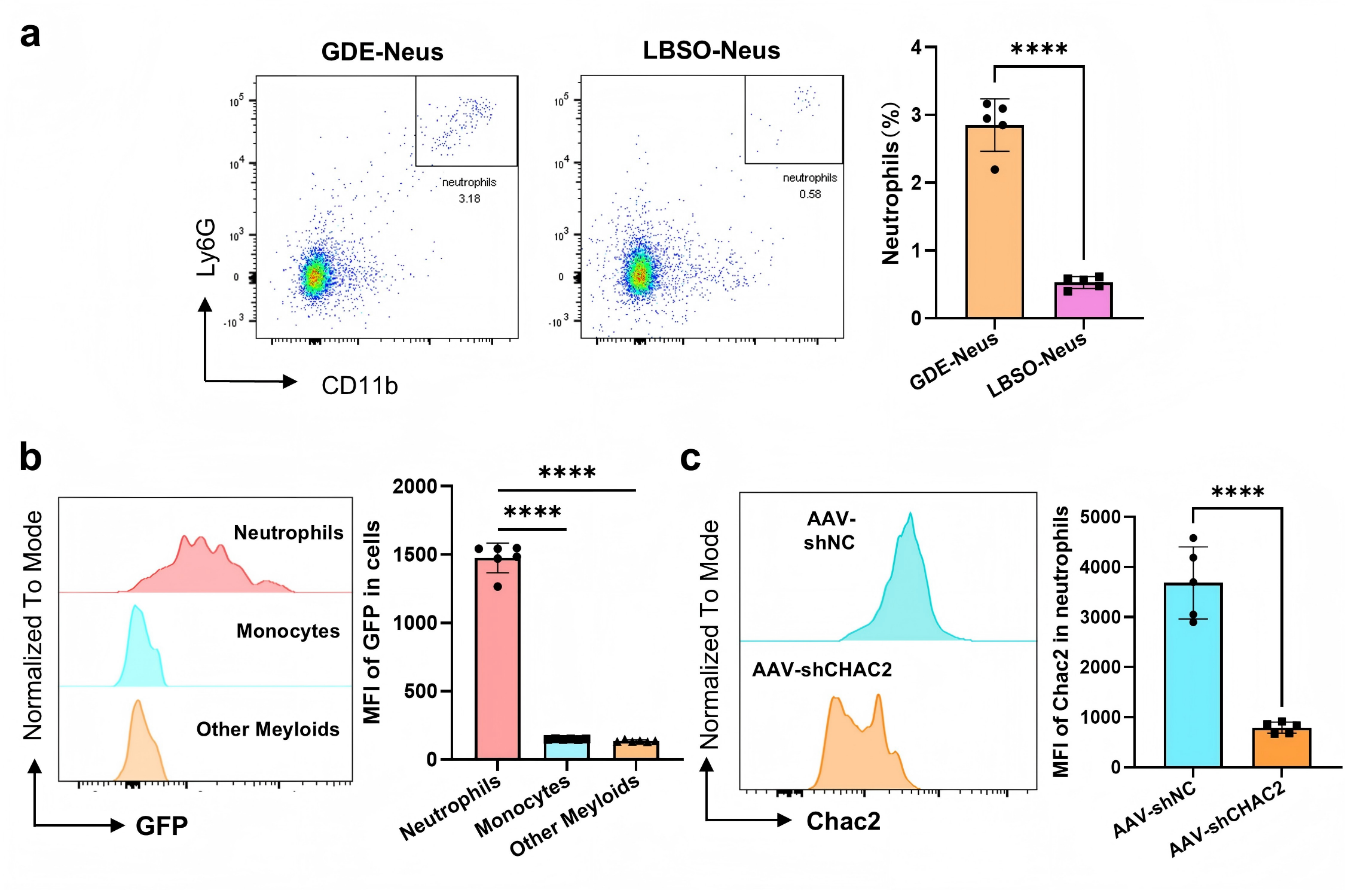


**Fig. S9 Glutathione modulation and Chac2 knockdown regulate pancreatic neutrophil accumulation and Chac2 expression in vivo**

**(a)** Representative flow cytometry dot plots showing the percentage and the statistic frequencies of pancreatic neutrophils (CD45^+^CD11b⁺Ly6G⁺) in mice receiving adoptive transfer of GDE-Neus and L-BSO-Neus. **(b)** Flow cytometric analysis of the expression of GFP in neutrophils (CD11b⁺Ly6G⁺), monocytes (CD11b⁺Ly6G⁻F4/80⁺) and other myeloid cells (CD11b⁺Ly6G⁻F4/80⁻) obtained from the BM after AAV-shCHAC2 intraosseous injection. **(c)** Representative flow cytometry histograms of intracellular Chac2 staining and modified fluorescence intensity quantification in BM neutrophils (CD11b⁺Ly6G⁺) isolated from mice injected intraosseously with AAV-shNC or AAV-shChac2. Data are presented as the mean ± SD. *P* values were calculated using two-tailed Student's t test (a, c)) and one-way ANOVA followed by Tukey’s multiple comparisons test (b);**P* <0.05, ***P* <0.01, ****P* <0.001, *****P* <0.0001.

**Table S1: The primers used for RT-qPCR.**

| **Gene** | **Primer sequence (5' to 3') -Forward** | **Primer sequence (5' to 3')-Reverse** |
| --- | --- | --- |
| *Gapdh* | CCATCACCATCTTCCAGG | AGACTCCACGACATACTCA |
| *Chac2* | GTGACCCTTGTTGAAGATCCTGG | GACTGTCGTAGTTCTGTAGCCTC |
| *Chac1* | TGACCCTCCTTGAAGACCGTGA | AGTGTCATAGCCACCAAGCACG |
| *Gsr* | GTTTACCGCTCCACACATCCTG | GCTGAAAGAAGCCATCACTGGTG |
| *Gclc* | ACACCTGGATGATGCCAACGAG | CCTCCATTGGTCGGAACTCTAC |
| *Gclm* | TCCTGCTGTGTGATGCCACCAG | GCTTCCTGGAAACTTGCCTCAG |
| *Gss* | CCAGGAAGTTGCTGTGGTGTAC | GCTGTATGGCAATGTCTGGACAC |
| *Anpep* | CGAACACCGTTTATCTGGACCTG | AGCCCATCTGTAGAATCCAGCG |
| *Gstk1* | AAGCTCCTGAGACACCATCTCC | CTCTGGATGCTCCAAGTTCACG |
| *Gstm1* | TGTTTGAGCCCAAGTGCCTGGA | TAGGTGTTGCGATGTAGCGGCT |
| *Gsto1* | CGAACCTAAGGGAAGCGTTGGA | TTCCAGTCGCTGAAACCAAGGC |
| *Mgst1* | TGCGACCGCATTCCAGAGGATA | TCCACCTTCTCGTCAGTGCGAA |
| *Gpx4* | CCTCTGCTGCAAGAGCCTCCC | CTTATCCAGGCAGACCATGTGC |
| *Pgd* | CATCGCTGCAAAAGTGGGAACC | AGCCTCACAGATGAGCTGCATG |
| *Idh1* | CAGGCTCATAGATGACATGGTGG | CACTGGTCATCATGCCAAGGGA |
| *G6pdx* | GACCAAGAAGCCTGGCATGTTC | AGACATCCAGGATGAGGCGTTC |
| *GAPDH* | GTCTCCTCTGACTTCAACAGCG | ACCACCCTGTTGCTGTAGCCAA |
| *CHAC1* | GTGGTGACGCTCCTTGAAGATC | GAAGGTGACCTCCTTGGTATCG |
| *CHAC2* | ACATCACCAACTACAGCAGGCG | CTGGCAATCTGTAAGCAACACCC |
| *IL-6* | AGACAGCCACTCACCTCTTCAG | TTCTGCCAGTGCCTCTTTGCTG |
| *IL-1B* | CCACAGACCTTCCAGGAGAATG | GTGCAGTTCAGTGATCGTACAGG |
| *TNF-A* | CTCTTCTGCCTGCTGCACTTTG | ATGGGCTACAGGCTTGTCACTC |
